# Structural Characterization of Acidic Polysaccharides from *Scutellaria baicalensis* Georgi and Its Protection Against Acute Lung Injury

**DOI:** 10.3390/antiox15091056

**Published:** 2026-08-24

**Authors:** Shuang Liu, Jia Li, Mingkun Li, Yuzhang Mi, Jianlin Ke, Jinglei Wang, Hongjing Dong, Xiao Wang

**Affiliations:** 1 School of Pharmacy, Shandong University of Traditional Chinese Medicine, Jinan 250300, China; 2 Shandong Engineering Research Center for Innovation and Application of General Technology for Separation of Natural Products, Shandong Analysis and Test Center, Qilu University of Technology (Shandong Academy of Sciences), Jinan 250014, China; 3 Key Laboratory for Natural Active Pharmaceutical Constituents Research in Universities of Shandong Province, School of Pharmaceutical Sciences, Qilu University of Technology (Shandong Academy of Sciences), Jinan 250014, China

**Keywords:** acute lung injury, polysaccharides, structural properties, gut-lung axis, *Scutellaria baicalensis* Georgi

## Abstract

Acute lung injury (ALI) seriously impairs health and well-being. Although *Scutellaria baicalensis* Georgi (SBG) extract has been shown to alleviate ALI, the effects of SBG acidic polysaccharides on ALI have not been revealed. In this study, a homogeneous acidic polysaccharide (SBGP1) was purified and characterized, and its impact on ALI was evaluated. Our findings illustrated that the average molecular weight of SBGP1 was 40764.6 Da, and it was mainly composed of GalA (53.14%), Ara (26.99%), Gal (7.75%), Glc (5.95%), and Rha (6.17%). The main chain of SBGP1 consisted of →4)-*α*-Gal*p*A-6-OMe-(1→ and →4)-*α*-Gal*p*A-(1→. Furthermore, SBGP1 exhibited a significant alleviating effect on ALI, as evidenced by decreased levels of TNF-*α*, IL-1*β*, IL-6, GSSG, and MDA, and increased levels of IL-4, IL-10, GSH, and SOD. Mechanistically, SBGP1’s role in alleviating ALI may be associated with the activation of the Rap1 signaling pathway. SBGP1 improved intestinal homeostasis, manifested as increased abundance of beneficial bacteria (*Lactobacillus* and *norank_f_Muribaculaceae*) and decreased abundance of harmful bacteria (*Adlercreutzia*, *Ligilactobacillus*, *Massiliomicrobiota*, and *Mucispirillum*). Interestingly, SBGP1-derived *Lactobacillus johnsonii* and propionic acid evidently improved the inflammatory response and oxidative stress in ALI mice. Overall, this study provides novel insights into SBGP1 as a potential therapeutic option for ALI.

## 1. Introduction

Acute lung injury (ALI) is a fast-evolving lung condition that arises from diverse non-cardiogenic causes, whether thoracic or systemic in origin. It is characterized by severe inflammation, impaired gas exchange, and acute respiratory difficulty [1]. In the United States, ALI affects roughly 78.9 per 100,000 individuals aged 15 years and older, with a corresponding fatality rate of 38.5% [2]. Additionally, a previous study in pediatric intensive care units (PICUs) revealed that among 1738 children younger than 15 years, the incidence of ALI was 51.2%, with a mortality rate of 7.4% [3]. ALI seriously affects the physical and mental health of patients. Modern pharmacological research has explored the pathogenesis of ALI and found that ALI is commonly accompanied by overexpression of pro-inflammatory cytokines such as tumor necrosis factor-alpha (TNF-*α*), interleukin-6 (IL-6), and interleukin-1 beta (IL-1*β*), as well as oxidative stress imbalance, as evidenced by increased levels of oxidized glutathione (GSSG) and malondialdehyde (MDA), and decreased levels of glutathione (GSH) and superoxide dismutase (SOD) [4]. Furthermore, these pro-inflammatory cytokines are released into the systemic circulation, inducing gut microbiota disorder, disruption of tight junctions, and subsequent gut barrier impairment. Increased intestinal permeability further increases the risk of intestinal infections and disrupts the lung immune microenvironment through blood circulation, lymphatic reflux, and neuroendocrine pathways, exacerbating ALI, resulting in a vicious cycle [5]. Currently, the management of inflammatory disorders predominantly relies on clinical agents such as ulinastatin, nitric oxide synthase inhibitors, angiopoietin-1, and glucocorticoids. However, these drugs may cause secondary lung injury, osteonecrosis, and gastrointestinal disorders [3]. Therefore, there is an urgent demand for more effective therapeutic options to enhance or supplement existing treatment strategies, especially those from natural products.

Polysaccharides are high-molecular-weight carbohydrates constructed from monosaccharide molecules linked by glycosidic bonds via dehydration condensation, and are conventionally grouped into neutral and acidic subclasses [6]. Acidic polysaccharides, which possess acidic substituents including sulfate and uronic acid moieties, occur ubiquitously across plant, animal, and microbial kingdoms [7]. Compared with neutral polysaccharides, acidic polysaccharides generally exhibit stronger biological activities, which are associated with their structural properties, including uronic acid content, molecular size, and monosaccharide composition. Numerous studies have documented that acidic polysaccharides possess a range of biological activities, such as anti-inflammatory [8], antioxidant [9], and gut microbiota-modulating [10] properties. Furthermore, acidic polysaccharides have been shown to exert beneficial effects on ALI [11]. Consequently, acidic polysaccharides are potential candidates for alleviating ALI.

*Scutellaria baicalensis* Georgi (SBG), a perennial medicinal herb, is found throughout East Asia and parts of Eurasia, with its range covering China, Japan, Korea, Mongolia, and Russia [12]. Its constituents include a variety of compounds, notably flavonoids, polysaccharides, and essential oils [13]. Among these, polysaccharides are the main chemical components in SBG, accounting for 7.1%, and are often wasted in the industrial production of baicalin [12,14]. Recent studies have indicated multiple bioactivities of SBG polysaccharides. For instance, SBG polysaccharides could ameliorate colitis by suppressing NF-κB signaling and NLRP3 inflammasome activation as well as restoring disorders in gut microbiota [15,16]. Xiang et al. [12] demonstrated that SBG polysaccharides possess immunomodulatory activity by promoting RAW264.7 macrophage proliferation, enhancing phagocytic ability, and stimulating nitric oxide and cytokine production. In addition, SBG polysaccharides have antioxidant and hypoglycemic activities [17]. However, acidic polysaccharides from SBG have not been fully explored.

The major aim of this study is to isolate and purify acidic polysaccharides from SBG and evaluate their effects in a mouse model of LPS-induced ALI. This study will contribute to the rational development and utilization of SBG acidic polysaccharides, as well as provide a novel therapeutic strategy for treating ALI.

## 2. Materials and Methods

### 2.1. Materials and Reagents

Dried SBG samples were collected from Laiwu, Shandong, China, and the authenticity of the samples was identified by Professor Jia Li, Shandong University of Traditional Chinese Medicine. Deionized water was prepared using a Millipore Direct-Q 8UV-R system (Merck, Darmstadt, Germany). All other reagents were of analytical grade.

### 2.2. Extraction of Polysaccharides from SBG

Dried SBG (200 g) was transferred to a round-bottom flask and extracted with 2000 mL of distilled water under reflux for 120 min. The resulting solution was vacuum-filtered, and the filtrate was evaporated at 55 °C under reduced pressure. Polysaccharides were then precipitated by adding ethanol to a final concentration of 80% (*v*/*v*) and allowing the mixture to stand overnight at 4 °C. After centrifugation, the pellet was redissolved in distilled water (50 mL/g). Proteins were eliminated through seven cycles of the Sevag procedure (chloroform/n-butanol, 4:1, *v*/*v*), and residual organic solvents were stripped off via rotary evaporation at 55 °C. The resultant aqueous phase was dialyzed against deionized water using a 3.5 kDa cutoff membrane and lyophilized to afford crude SBG polysaccharides. For further fractionation, 10 g of this crude material was reconstituted in 50 mL of deionized water and loaded onto a DEAE-52 Sepharose column (3.5 × 60 cm). Stepwise elution was performed with 5 column volumes each of deionized water and increasing concentrations of NaCl (0.1, 0.2, 0.3, 0.4, and 0.5 mol/L), with elution profiles monitored by the phenol–sulfuric acid assay. The fraction eluted at 0.1 mol/L NaCl was collected, concentrated, and freeze-dried. A 0.5 g aliquot of this fraction was then applied to a Sephadex G-100 gel filtration column and eluted with deionized water. The major peak fraction was collected, dialyzed (3.5 kDa cutoff), and lyophilized, yielding the purified acidic polysaccharide designated SBGP1.

### 2.3. Structural Properties of SBGP1

#### 2.3.1. Molecular Weight (Mw) Distribution Determination

Molecular mass assessment of SBGP1 was performed by HPGPC-RID (Hanon Advanced Technology Group Co., Ltd., Jinan, China) using a set of glucan reference standards (5000, 150,000, 270,000, 410,000, and 670,000 Da). The system was equipped with two Shodex OH-pak columns (SB-805 and SB-804, 300 mm × 8 mm, Showa Denko K.K., Tokyo, Japan) in tandem and an RI-201H detector (Showa Denko, Tokyo, Japan). The sample (15 mg in 1 mL water) was 0.22 μm filtered prior to injection of a 30 μL aliquot, and the mobile phase (0.1 mol/L NaCl) was delivered at a constant flow rate of 1.0 mL/min.

#### 2.3.2. Monosaccharide Components Analysis

Dried SBGP1 (5 mg) was hydrolyzed in 4 mL of 2 mol/L trifluoroacetic acid (TFA; Kemiou Chemical Reagent Co., Ltd., Tianjin, China) at 120 °C for 2 h. Subsequently, the hydrolysates were concentrated using a rotary evaporator (EYELA, Tokyo, Japan) and washed three times with methanol (Fuyu Chemical, Tianjin, China). The resulting solution was reconstituted in 1 mL of deionized water and filtered through a 0.22 μm membrane prior to chromatographic analysis.

Ion chromatography was carried out on an ICS5000 system (Thermo Fisher Scientific, Waltham, MA, USA), which was fitted with a Dionex CarboPac PA-20 anion-exchange column (3.0 × 150 mm, 10 μm;, Thermo Scientific, Waltham, USA). The column oven was set to 30 °C during the entire run. Elution employed a binary mobile phase consisting of (A) 0.1 mol/L NaOH and (B) 0.1 mol/L NaOH supplemented with 0.2 mol/L NaAc, delivered at 0.5 mL/min. The gradient timetable was programmed as follows: 5% B at 0 min, 20% B at 30 min, 40% B at 30.1 min, held at 40% B until 45 min, then returned to 5% B at 45.1 min and maintained until 60 min. External calibration was adopted for monosaccharide quantification.

#### 2.3.3. Fourier-Transform Infrared Spectroscopy (FT-IR) Analysis

Dried SBGP1 was homogenized with potassium bromide (KBr) powder. Subsequently, FT-IR spectroscopic analysis was conducted using a Fourier-transform infrared spectrometer (Thermo Scientific, Waltham, USA) to acquire the FT-IR spectrum for structural characterization.

#### 2.3.4. Scanning Electron Microscope (SEM) Analysis

SEM analysis was performed using a SUPRA 55 thermal field-emission scanning electron microscope (Carl Zeiss, Jena, Germany). Before analysis, SBGP1 was coated with metal by sputtering with either gold or platinum, followed by visualization via secondary electron signal detection.

#### 2.3.5. Methylation Analysis

Methylation analysis of SBGP1 commenced with dissolving 5 mg of the sample in 1 mL of water, followed by addition of 1 mL of 1-cyclohexyl-2-morpholinoethylcarbodiimide methyl p-toluenesulfonate (2 h reaction). After introducing 1 mL of imidazole, the solution was equally divided; each half received NaBH_4_ and NaBD_4_ (both at 30 mg/mL, 1 mL each) for a 3 h reduction. The reaction was halted with 100 μL of glacial acetic acid, and the mixture was dialyzed (48 h) and freeze-dried. A 2 mg sample of the lyophilizate was taken up in 0.5 mL of DMSO containing 1 mg NaOH. After 30 min, 50 μL of CH_3_I was added for 1 h methylation. The product was partitioned between 1 mL water and 2 mL dichloromethane, vortexed, and centrifuged; the aqueous phase was discarded after three washings, and the organic extract was dried under N_2_. Hydrolysis of the permethylated material employed 100 μL of 2 mol/L TFA at 120 °C for 90 min, with the acid removed by evaporation at 30 °C. Reduction was then performed by adding 50 μL of 2 mol/L ammonia and 50 μL of 1 mol/L NaBD_4_, allowing 150 min at room temperature, and quenching with 20 μL acetic acid. Following methanol rinses (250 μL, ×3) and nitrogen drying, acetylation was carried out with 250 μL acetic anhydride for 2.5 h at 100 °C. The final product was diluted with 1 mL water, equilibrated for 10 min, and extracted into 500 μL dichloromethane; the organic phase was washed three times with water, centrifuged, and collected for instrumental analysis.

Derivatized samples were subjected to GC-MS on an Agilent 6890A-5975C system (Thermo Fisher Scientific) equipped with a TG-200MS column (30 m × 0.25 mm × 0.25 mm). The mass spectrometer was operated with an ion source at 200 °C, a quadrupole at 110 °C, an ionization energy of 50 eV, a transfer line temperature of 210 °C, and scanning from *m*/*z* 50 to 350. Chromatographic separation employed high-purity helium as the carrier gas at 1.5 mL/min, with a split ratio of 10:1 and a 1 μL injection. The thermal gradient started at 150 °C (1 min hold), rose to 210 °C at 2 °C/min (2 min hold), and continued to 240 °C at the same ramp rate (3 min hold).

#### 2.3.6. Nuclear Magnetic Resonance (NMR) Analysis

SBGP1 without carboxyl reduction (45 mg) was dried prior to dissolution in 0.5 mL of deuterium oxide (D_2_O, 99% purity). NMR analysis was subsequently performed at 25 °C using a Bruker Avance III HD 600 MHz Nuclear magnetic resonance spectrometer (Bruker, Karlsruhe, Germany) to obtain ^1^H NMR, ^13^C NMR, and 2D NMR spectra (COSY, HSQC, and HMBC).

### 2.4. Effects of SBGP1 on LPS-Induced ALI

#### 2.4.1. Animals and Experimental Design

Male C57BL/6J mice (22 ± 1 g) were purchased from Jinan Pengyue Laboratory Animal Breeding Co., Ltd. (Jinan, China) and maintained under standard laboratory conditions, including a temperature of 22 ± 2 °C, 55 ± 15% relative humidity, and a 12 h light/dark cycle. Animals had free access to food and water. All procedures were conducted in accordance with the guidelines approved by the Institutional Animal Ethics Committee of Shandong Academy of Chinese Medicine (Approval ID: SDZYY20260112004).

Experiment 1: Mice were randomly divided into five groups: control group (C, *n* = 6), ALI group (M, *n* = 6), dexamethasone group (5 mg/kg, Y, *n* = 6), low dose of SBGP1 group (150 mg/kg, SBGP1-L, *n* = 6), and high dose of SBGP1 group (300 mg/kg, SBGP1-H, *n* = 6). Prior to drug intervention, except for in the C group, mice were treated with lipopolysaccharide (LPS, 5 mg/kg) by intratracheal instillation for 3 consecutive days [18]. Starting from the 4th day of administration, mice were euthanized after 7 days, and bronchoalveolar lavage fluid (BALF), serum, intestinal, lung, and fecal samples were collected for subsequent analysis. The lung index was determined using the formula: lung index = lung weight (g)/body weight (g) × 100%.

Experiment 2: Mice were randomly divided into four groups: control group (C, *n* = 6), ALI group (M, *n* = 6), pseudo germ-free mice group (GM-K, *n* = 6), and SBGP1 fecal microbiota transplantation group (FMT-SBGP1, *n* = 6). Before drug intervention, except for in the C group, mice were treated with LPS (5 mg/kg) by intratracheal instillation for 3 consecutive days. After LPS intervention for 3 days, mice were administered their respective treatments for 7 days and then euthanized. BALF, serum, intestinal tissue, lung, and fecal samples were collected for subsequent analysis.

Experiment 3: Mice were randomly divided into four groups: control group (C, *n* = 6), ALI group (M, *n* = 6), *Lactobacillus johnsonii* (1 × 10^9^ CFU/mL)-treated group (LJ), and pasteurized *Lactobacillus johnsonii* (1 × 10^9^ CFU/mL)-treated group (LJS). Mice in the LJ and LJS groups were administered 100 μL of an antibiotic cocktail (1 g/L neomycin, 1 g/L ampicillin, 1 g/L metronidazole, 0.5 g/L vancomycin) daily for 7 d before microbiota transplantation. Subsequently, fecal microbiota transplantation was performed daily for 7 days. After 7 days, mice were killed, and BALF, serum, intestinal tissue, lung and feces samples were collected.

Experiment 4: Mice were randomly divided into three groups: control group (C, *n* = 6), ALI group (M, *n* = 6), and propionic acid group (100 mg/kg, PA, *n* = 6). Before drug intervention, except for in the C group, mice were treated with LPS (5 mg/kg) by intratracheal instillation for 3 consecutive days. After drug administration for 7 days, mice were euthanized, and BALF, serum, intestinal tissue, lung and feces samples were collected for subsequent analysis.

#### 2.4.2. Biochemical Analyses

Pro-inflammatory cytokines, including TNF-*α*, IL-1*β*, IL-6, IL-4 and IL-10, were determined using enzyme-linked immunosorbent assay (ELISA) kits (NeoBioscience Technology Co., Ltd., Shenzhen, China). The levels of oxidative stress indices such as MDA, GSSG, and GSH were evaluated with commercially available assay kits (Shanghai Enzyme Linked Biotechnology Co., Ltd., Shanghai, China). The expression of SOD was measured with assay kits from Shanghai Beyotime Biotechnology Co., Ltd. (Shanghai, China).

#### 2.4.3. Lung and Intestine Histopathological Analysis

The histopathological evaluation of lung and intestinal tissue specimens was conducted using hematoxylin and eosin (H&E) staining. Briefly, fresh tissues were fixed in 4% paraformaldehyde and embedded in paraffin. Sections were cut using a Leica 531CM-Y43 rotary microtome (Leica, Wetzlar, Germany) and deparaffinized with xylene. Subsequently, the sections were immersed in absolute ethanol for 5 min, 95% ethanol for 5 min, 85% ethanol for 5 min, then transferred to 75% ethanol for 5 min, and finally soaked in distilled water for 5 min. The sections were stained with hematoxylin for 3 min and eosin for 20 s. Dehydration was carried out using a graded ethanol series: 75% ethanol for 10 s, 80% ethanol for 10 s, 95% ethanol for 20 s, and absolute ethanol for 90 s. The sections were then treated with xylene to clear them. Finally, the sections were examined under a Leica DM IL LED fluorescence inverted microscope (Leica, Wetzlar, Germany).

#### 2.4.4. Transcriptomic Analysis

Total RNA was extracted from lung tissues of mice in the C, M, and SBGP1 groups using TRIzol^®^ Reagent (Thermo Scientific, Waltham, USA). The RNA purification, reverse transcription, library construction and sequencing were performed at Shanghai Majorbio Bio-pharm Biotechnology Co., Ltd. (Shanghai, China). Low-quality bases and contaminants in raw reads were removed using FASTP (v0.19.5). High-quality clean reads were then mapped to the mouse reference genome (Ensembl) using HISAT2 (v2.1.0). The R package “limma” was used to identify differentially expressed genes (DEGs) based on criteria such as log_2_FC ≥ 0.25 and adjusted *p* < 0.05. Enrichment analysis of Gene Ontology (GO) and Kyoto Encyclopedia of Genes and Genomes (KEGG) for DEGs was carried out using the “clusterProfiler” R package (v4.4.0).

#### 2.4.5. Gut Microbiota Analysis

For gut microbiota analysis, fecal DNA from C, M, and SBGP1-H mice was extracted (E.Z.N.A. Soil DNA Kit, Omega Bio-tek, Norcross, USA) and the V3-V4 region of 16S rRNA was amplified using primers 338F/806R. The 20 μL PCR system contained 4 μL of 5× Fast Pfu buffer, 2 μL of 2.5 mM dNTPs, 0.8 μL of each primer (5 μM), 0.4 μL of Fast Pfu polymerase, and 10 ng of DNA. Thermal cycling: 95 °C/3 min; 27 × (95 °C/30 s, 55 °C/30 s, 72 °C/45 s); 72 °C/10 min; hold at 4 °C. Amplicons were purified (PCR Clean-Up Kit, YuHua, Shanghai, China) and sequenced on the Illumina NextSeq 2000 platform (Illumina, San Diego, USA). R software was used for alpha/beta diversity and community composition, LEfSe for discriminative genera identification, and Tax4Fun2 for functional annotation based on OTUs.

#### 2.4.6. Short-Chain Fatty Acids (SCFAs) Metabolomics Analysis

The content of SCFAs was quantified according to a previously described protocol [19]. Briefly, a fecal sample (20 mg) was mixed with propanol at a ratio of 2:1 (w/w), and then extracted using 50% acetonitrile (200 μL). After centrifuging at 10,000 rpm for 10 min, the supernatant was collected. Then, 40 μL of the supernatant was subjected to derivatization with 20 μL of 3-nitrophenylhydrazine hydrochloride (200 mmol/L) and 20 μL of N-(3-dimethylaminopropyl)-N′-ethylcarbodiimide (200 mmol/L). Before chromatographic analysis, 0.92 mL of 10% acetonitrile was added to dilute the derivatized solution.

An ultra-performance liquid chromatography system coupled with a Xevo TQ-XS triple quadrupole mass spectrometer (Waters Corp., Milford, MA, USA) was used for the quantification of SCFAs. Specifically, 5 μL the derivatized sample solution was loaded onto a Waters ZORBAX SB-C18 column (2.1 × 100 mm, 1.8 μm), and eluted with mobile phases A (0.1% formic acid in water) and B (acetonitrile) at a flow rate of 0.3 mL/min using the following gradient: 5% B (0 min), 15% B (1 min), 30% B (5–7 min), 45% B (9 min), 100% B (11–12 min), 5% B (12.1–15 min). Mass spectrometric conditions: capillary voltage 2.5 kV, desolvation temperature 450 °C, nebulization pressure 7.0 bar, desolvation gas flow 15 L/min, ion energy1 0.3, ion energy2 0.9, cone voltage 30V, cone gas flow 150 L/hr, desolvation gas flow 900 L/hr. High-purity N_2_ (99.99%) was used as desolvation and collision gas.

#### 2.4.7. Prediction of Mechanism of PA on ALI Based on Network Pharmacology

To predict therapeutic targets and disease-related genes, we utilized the SuperPRED platform (https://prediction.charite.de/, accessed on 2 February 2026) for compound target forecasting and the GeneCards database (https://www.genecards.org/, accessed on 2 February 2026) with the query “lung inflammation” for disease gene retrieval. The Venny tool (https://bioinfogp.cnb.csic.es/tools/venny/, accessed on 2 February 2026) was then employed to intersect the two gene sets and identify common candidates. These overlapping targets underwent functional annotation via Gene Ontology (GO) and Kyoto Encyclopedia of Genes and Genomes (KEGG) pathway enrichment analyses using the DAVID database (https://davidbioinformatics.nih.gov/, accessed on 2 February 2026), and their inter-relationships were further explored by constructing a PPI network via STRING (https://cn.string-db.org/, accessed on 2 February 2026).

#### 2.4.8. Immunohistochemical Analysis

Immunohistochemical staining for RAP1A was conducted on the lung tissue sections. Specifically, after the paraffin sections were baked at 65 °C for 60 min for dewaxing, they were sequentially hydrated with xylene and gradient ethanol (100% → 95% → 85% → 75% → 50%) and finally soaked in ultrapure water for 5 min. The slices were then placed in sodium citrate antigen retrieval solution, and antigen retrieval was performed by microwave heating. After being allowed to cool naturally, the slices were washed with PBS. Endogenous peroxidase blocking solution was added dropwise to the slices, and blocking was carried out at room temperature for 10 min, followed by washing with PBS. Goat serum blocking solution was added dropwise, and blocking was performed at 37 °C for 30 min. After the serum was removed, primary antibody was added, and the slices were incubated overnight at 4 °C, then washed with PBS. Secondary antibody (RAP1A, diluted 1:200) was added and incubated at room temperature for 40 min, followed by washing with PBS. DAB color reagent was used for color development, with the reaction time controlled under a microscope, and the reaction was terminated with PBS. Counterstaining was performed with hematoxylin for 30 s, followed by differentiation with differentiation buffer for 3 s, and then bluing was carried out under running water for 5 min. After gradient ethanol dehydration and xylene clearing, the selected areas were captured using a Leica DM IL LED microscope (Leica Microsystems Trading Co., Ltd., Shanghai, China), and quantification was performed using ImageJ software (v1.54p) based on uniform threshold settings.

### 2.5. Statistical Analysis

All data are presented as mean ± standard deviation (SD). Statistical analyses were performed using R software (version 4.4.0). For comparisons among multiple groups, one-way analysis of variance (ANOVA) was conducted using the aov() function, followed by Tukey’s honestly significant difference (HSD) post hoc test for all pairwise comparisons. In the gut microbiota analysis, Benjamini–Hochberg (BH) false discovery rate (FDR) correction was applied to correct for multiple comparisons. Adjusted *p*-values < 0.05 were considered statistically significant to control the expected proportion of false positives.

## 3. Results

### 3.1. Structural Characterization of SBGP1

#### 3.1.1. *M*w and Monosaccharide Composition

**Figure 1:**
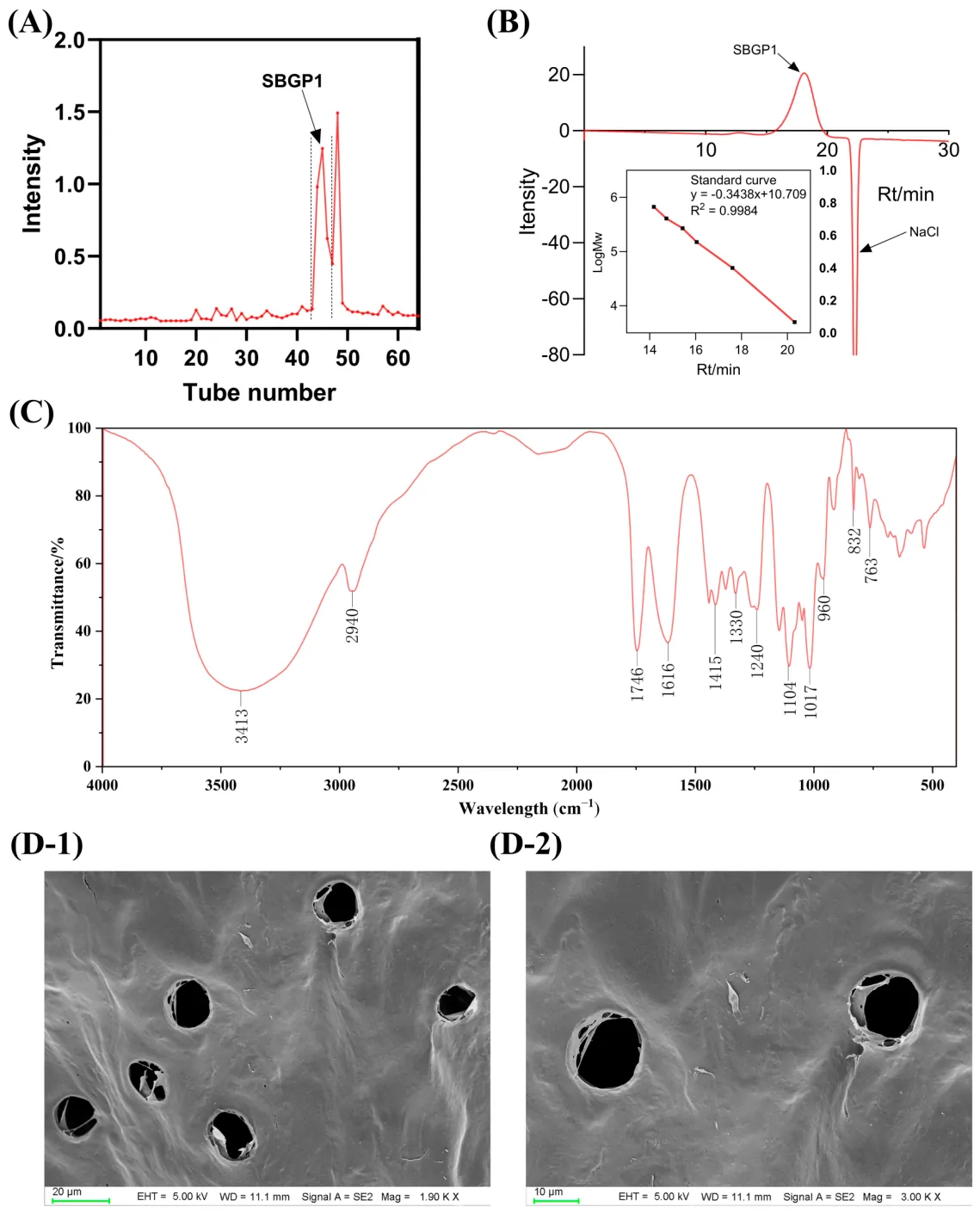
Elution curve of Sephadex G-100 column (**A**); molecular weight distribution (**B**); FT-IR spectra (**C**); SEM images: 1900× (**D-1**) and 3000× (**D-2**).

SBG crude polysaccharides were loaded onto a DEAE-52 cellulose column, and the fraction eluted by 0.1 mol/L NaCl was obtained (Figure S1A). Subsequently, the fraction was further purified using a Sephadex G-100 column, and the purified polysaccharide was named SBGP1 (Figure 1A). Molecular weight distribution indicated that SBGP1 was a homogeneous polysaccharide with an average *M*w of 40764.6 Da (Figure 1B). Monosaccharide composition analysis showed that SBGP1 was primarily composed of GalA (53.14%), Ara (26.99%), Gal (7.75%), Glc (5.95%) and Rha (6.17%) (Figure S1B).

#### 3.1.2. FT-IR Spectra and Morphological Characteristics

**Figure 2:**
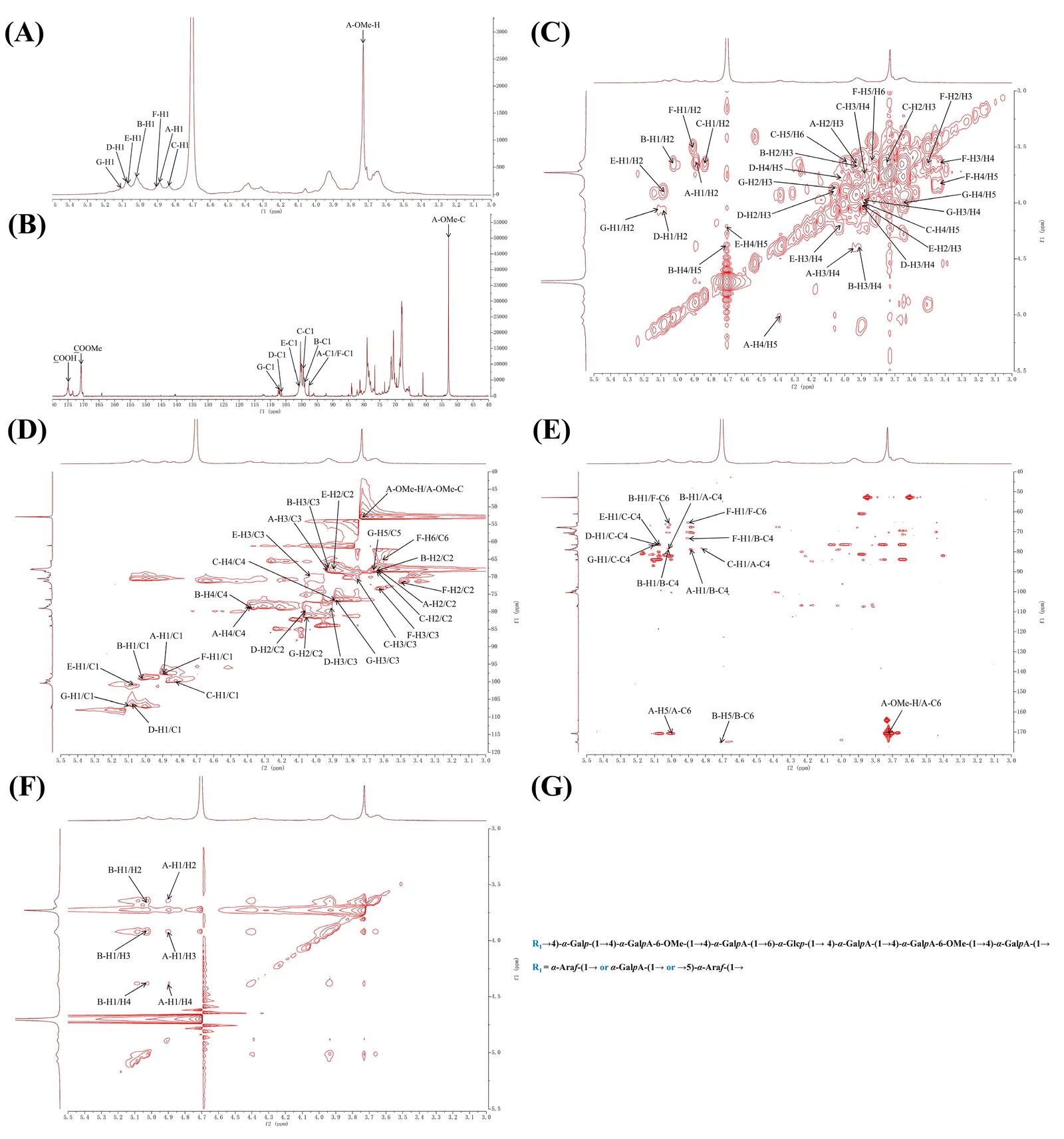
NMR spectra: ^1^H (**A**); ^13^C (**B**); H-H COSY (**C**); HSQC (**D**); HMBC (**E**); NOESY (**F**); proposed structural fragment of SBGP1 (**G**).

To gain further insight into the structural features of SBGP1, its FT-IR spectrum was acquired (Figure 1C). A broad absorbance centered at 3413 cm^−1^ arose from O-H stretching vibrations of hydroxyl groups, whereas the band at 2940 cm^−1^ corresponded to C-H stretching [9]. The absorptions appearing at 1746 cm^−1^ and 1616 cm^−1^ were assignable to the asymmetric stretching of methyl-esterified carboxyl moieties (COO-R) and the stretching of ionic carboxylate groups (COO^−^), respectively, which aligns with the GalA content revealed by monosaccharide analysis [20]. The signal at 1415 cm^−1^ reflected the stretching of -OCH_3_ groups, pointing to methyl esterification of GalA [21]. A band at 1240 cm^−1^ was indicative of C-O stretching vibrations [22]. The absorptions observed at 1104 and 1017 cm^−1^ originated from C-O bending within C-O-H or C-O-C linkages, suggesting the presence of a pyranose ring configuration, which is in agreement with the pyranose monosaccharides (GalA, Gal, and Glc) identified by compositional analysis [23]. The peak at 960 cm^−1^ arose from O-H bending in carboxylic acids, further corroborating the GalA detection [24]. Additionally, the band at 832 cm^−1^ pointed to an α-type glycosidic linkage, while the signal at 763 cm^−1^ corresponded to the characteristic symmetric ring-breathing vibration of the pyranose ring [25,26]. Finally, SEM imaging (Figure 1(D-1,D-2)) revealed that SBGP1 exhibited a smooth surface with conspicuous pores.

#### 3.1.3. Methylation Analysis

SBGP1 underwent methylation, hydrolysis, and acetylation to yield PMAAs, which were subsequently analyzed by GC-MS (Figure S2). According to Table 1, 14 PMAA derivatives were assigned by matching their relative retention times and mass spectral fragmentation patterns against the PMAAs reference database. Briefly, the 1,4-linked-Gal*p*A (Rt 17.11 min) exhibited diagnostic ions at *m*/*z* 87, 101, 102, 115, 118, 129, 133, 162, 175, and 235. The 1,4-linked-Gal*p* (Rt 17.10 min) showed diagnostic ions at *m*/*z* 87, 99, 102, 113, 118, 129, 131, 162, 173, and 233. The T-linked-Gal*p*A was identified by ions at *m*/*z* 73, 89, 102, 118, 131, 147, 162, 163, and 207. The methylation analysis suggested that SBGP1 was predominantly composed of terminal residues (T-linked-Ara*f*), unsubstituted residues (1,4-linked-Gal*p*A, 1,4-linked-Gal*p*, and 1,6-linked-Glc*p*), and monosubstituted residues (1,3,4-linked-Gal*p*A, 1,2,5-linked-Ara*f*, and 1,2,4-linked-Rha*p*). Notably, a high proportion of sugar residues, such as 1,4-linked-Gal*p*A, 1,4-linked-Gal*p* and 1,6-linked-Glc*p*, may constitute the main chain of SBGP1.

#### 3.1.4. NMR Analysis

Polysaccharides were characterized by high molecular weight and complex structure, causing an overlap in proton signals at *δ* 3.0–5.5 ppm. Consequently, 1D (^1^H and ^13^C) and 2D (COSY, HSQC, HMBC, and NOESY) NMR spectra were acquired to reveal the proposed structural fragment of SBGP1 (Figure 2).

The anomeric proton signals of glycosidic residues commonly appeared at *δ* 4.3–5.5 ppm in the ^1^H spectra, while other proton signals were distributed in the range of *δ* 3.0–4.3 ppm. As depicted in Figure 2A, a total of seven anomeric proton signals were found at *δ* 4.89, 5.02, 4.83, 5.08, 5.07, 4.90 and 5.11 ppm, and were assigned to glycosidic residues A, B, C, D, E, F and G, respectively. In the HSQC spectra, a cross-peak at *δ* 4.89/97.68 ppm was observed and assigned to H1/C1 of *α*-Gal*p*. Cross-peak signals at *δ* 4.89/3.63, 3.63/3.93, 3.93/4.40, and 4.40/5.01 ppm were detected in the COSY spectra, indicating H2, H3, H4, and H5 of residue A were *δ* 3.63, 3.93, 4.40, and 5.01 ppm, respectively. The correlations of *δ* 4.89/97.68, 3.63/67.75, 3.93/68.46, 4.40/78.40, and 5.01/70.49 ppm observed in the HSQC spectra should be separately attributed to H1/C1, H2/C2, H3/C3, H4/C4, and H5/C5 of glycosidic residue A. In the HMBC spectra, a cross-peak at *δ* 5.01/170.80 ppm was observed, suggesting the C6 signal of glycosidic residue A was *δ* 170.80 ppm. Due to the chemical shift in C4 of glycosidic residue A towards the low field direction, it was identified as 1,4-linked-Gal*p*A. Additionally, the peak signal at *δ* 3.73/52.86 ppm found in the HSQC spectra represented the presence of methyl ester (-COOMe), and the cross-peak signal at *δ* 3.73/170.80 ppm detected in the HMBC spectra further demonstrated the methylation of glycosidic residue A at C6. Therefore, glycosidic residue A was ultimately identified as →4)-*α*-Gal*p*A-6-OMe-(1→.

For glycosidic residue B, a cross-peak signal at *δ* 5.02/99.03 ppm was detected in the HSQC spectra, indicating the C1 signal of glycosidic residue B was *δ* 99.03 ppm. Multiple cross-peaks at *δ* 5.02/3.65, 3.65/3.92, 3.92/4.38, and 4.38/4.71 ppm were observed in the COSY spectra, suggesting H2, H3, H4, and H5 of glycosidic residue B were *δ* 3.65, 3.92, 4.38, and 4.71 ppm, respectively. The correlations of *δ* 5.02/99.03, 3.65/67.92, 3.92/68.07, 4.38/78.74, and 4.71/70.56 ppm observed in the HSQC spectra should be assigned to H1/C1, H2/C2, H3/C3, H4/C4, and H5/C5 of glycosidic residue B. In addition, the cross-peak signal at *δ* 4.71/174.90 ppm suggested the C6 signal of glycosidic residue B was *δ* 174.90 ppm, and methylation did not occur. The increase in chemical shift in C4 indicated that the C4 position was substituted. Based on the above analysis, glycosidic residue B was identified as →4)-*α*-Gal*p*A-(1→. Based on the relative signal intensity of anomeric hydrogen, the ratio of →4)-*α*-Gal*p*A-6-OMe-(1→ to →4)-*α*-Gal*p*A-(1→ was 19.99%: 30.67%. Additionally, the degree of methylation (DM) of SBGP1 was 39.46%.

In the analysis of glycosidic residue C, a cross-peak at *δ* 4.83/99.48 ppm was observed in the HSQC spectra, indicating the chemical shift in C1 of glycosidic residue C was *δ* 99.48 ppm. In the COSY spectra, cross-peak signals at *δ* 4.83/3.66, 3.66/3.75, 3.75/3.88, 3.88/4.00, and 4.00/3.65 ppm were observed and separately assigned to H1/H2, H2/C3, H3/C4, H4/C5, and H5/H6 of glycosidic residue C. Moreover, the correlations of *δ* 4.83/99.48, 3.66/68.02, 3.75/69.50, 3.88/76.53, 4.00/74.07, and 3.63/62.48 ppm indicated the chemical shifts in C1, C2, C3, C4, C5, and C6 of glycosidic residue C were *δ* 99.48, 68.02, 69.50, 76.53, 74.07, and 62.48 ppm, respectively. The downfield shift in the C4 of glycosidic residue C indicated substitution at the C4 position. Based on the above analysis, glycosidic residue C was identified as →4)-*α*-Gal*p*-(1→.

For glycosidic residue D, in the HSQC spectra, a cross-peak signal at *δ* 5.08/106.40 ppm was observed and assigned to H1/C1 of *α*-Ara*f*. On the basis of the correlations of H1/C1, H2/C2, H3/C3, H4/C4 and H5/C5 in the COSY and HSQC spectra, the cross-peak signals at *δ* 5.08/106.40, 4.06/80.10, 3.89/78.96, 4.02/80.98 and 3.77/61.09 ppm should be assigned to *α*-Ara*f*-(1→. Similarly, cross-peak signals at *δ* 5.11/4.05, 4.05/3.87, 3.87/4.00, and 4.00/3.65 ppm were observed in the COSY spectra, indicating H2, H3, H4, and H5 of glycosidic residue G were *δ* 4.05, 3.87, 4.00, and 3.65 ppm, respectively. In the HSQC spectra, the H1/C1, H2/C2, H3/C3, H4/C4, and H5/C5 of glycosidic residue G were confirmed to occur in signal regions of *δ* 5.11/106.92, 4.05/81.10, 3.87/76.68, 4.00/80.91, and 3.65/67.92 ppm. Furthermore, the relatively high chemical shift in C5 suggested substitution at this position. By integrating the above analysis, it was inferred that glycosidic residue G was →5)-*α*-Ara*f*-(1→.

Following a similar method, based on COSY, HSQC, and HMBC spectra, the H1/C1, H2/C2, H3/C3, H4/C4, H5/C5, and H6/C6 of glycosidic residue E were *δ* 5.07/100.93, 3.89/68.07, 4.03/69.87, 4.22/71.20, 4.71/70.67, and n.d./174.90 ppm, respectively. Meanwhile, the H1/C1, H2/C2, H3/C3, H4/C4, H5/C5, and H6/C6 of glycosidic residue F were *δ* 4.90/97.68, 3.50/71.37, 3.63/73.38, 3.43/69.50, 3.83/70.15, and 3.61/65.58 ppm. Therefore, glycosidic residues E and F were identified as *α*-Gal*p*A-(1→ and →6)-*α*-Glc*p*-(1→, respectively. All chemical shifts in protons/carbons for glycosidic residues of SBGP1 were presented in Table 2.

The HMBC spectrum was used to further elucidate the proposed structure of SBGP1. Cross-peak signals at *δ* 5.02/78.74 ppm and *δ* 4.90/65.58 ppm indicated the presence of repeating unit of →4)-*α*-Gal*p*A-(1→ and →6)-*α*-Glc*p*-(1→. The signal at *δ* 5.02/4.38 ppm in the NOESY spectrum also confirmed the repeating unit of →4)-*α*-Gal*p*A-(1→. The association of *δ* 4.89/78.74 ppm suggested the H1/C1 of →4)-*α*-Gal*p*A-6-OMe-(1→ connected with H4/C4 of →4)-*α*-Gal*p*A-(1→, while *δ* 5.02/78.40 ppm represented the connection between H1/C1 of →4)-*α*-Gal*p*A-(1→ and H4/C4 of →4)-*α*-Gal*p*A-6-OMe-(1→. Meanwhile, cross-peak signals at *δ* 4.89/4.38 ppm and *δ* 5.02/4.40 ppm also demonstrated the alternating connection between →4)-*α*-Gal*p*A-(1→ and →4)-*α*-Gal*p*A-6-OMe-(1→. The H1/C1 of →4)-*α*-Gal*p*-(1→ was connected to H4/C4 of →4)-*α*-Gal*p*A-6-OMe-(1→, as evidenced by the cross-peak signal at *δ* 4.83/78.40 ppm. Cross-peak signal at *δ* 5.02/65.58 ppm indicated the association of H1/C1 of →4)-*α*-Gal*p*A-(1→ and H6/C6 of →6)-*α*-Glc*p*-(1→. Cross-peak signal at *δ* 4.90/78.74 ppm suggested H1/C1 of →6)-*α*-Glc*p*-(1→ connected to H4/C4 of →4)-*α*-Gal*p*A-(1→. Additionally, multiple cross-peak signals at *δ* 5.08/76.56, *δ* 5.07/76.56, and *δ* 5.11/76.56 ppm indicated that *α*-Ara*f*-(1→, *α*-Gal*p*A-(1→, and →5)-*α*-Ara*f*-(1→ were all connected to H4/C4 of →4)-*α*-Gal*p*-(1→. All significant connections observed in the HMBC spectra of the anomeric protons/carbons of the glycosidic residues in SBGP1 were listed in Table 3. After a comprehensive analysis of all the evidence, the proposed structural fragment of SBGP1 was provided in Figure 2G.

### 3.2. SBGP1 Alleviated Inflammatory Response and Oxidative Stress in ALI Mice

**Figure 3:**
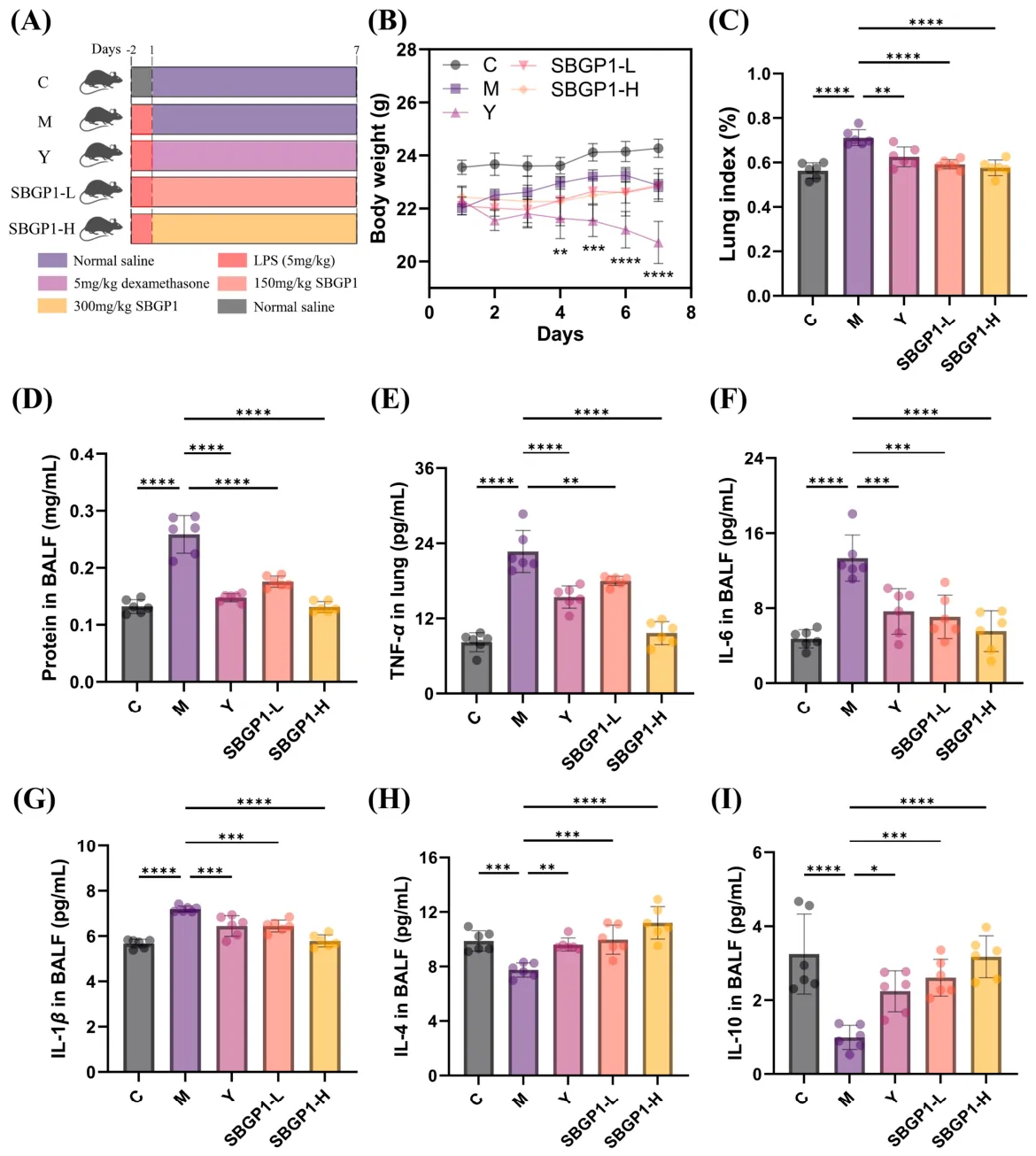
Schematic representation of the experimental plan (**A**); changes in body weight of ALI mice (**B**); changes in lung index (**C**); the relative levels of protein (**D**), TNF-*α* (**E**), IL-6 (**F**), IL-1*β* (**G**), IL-4 (**H**), and IL-10 (**I**) in BALF. All data represent *n* = 6 independent mice per group. * *p* < 0.05, ** *p* < 0.01, *** *p* < 0.001, and **** *p* < 0.0001.

To evaluate the effects of SBGP1 on ALI, an LPS-induced lung injury mouse model was established (Figure 3A). As depicted in Figure 3B, compared with the C group, LPS caused weight loss in mice. Subsequently, within the next 7 days, the body weight of the mice gradually increased. SBGP1 intake treatment did not significantly change the weight of mice, while the weight of mice in the Y group gradually decreased, which could be attributed to its side effects [29]. Lung index reflected the severity of lung injury. Compared with the C group, LPS significantly increased the lung index, while dexamethasone and low- and high-dose SBGP1 intake treatment evidently reversed the altered change (Figure 3C, *p* < 0.01). Low-dose and high-dose SBGP1 intake reduced lung index by 16.78% and 19.03%, respectively. Furthermore, the protein level in BALF was evidently increased in the M group compared to the C group (Figure 3D, *p* < 0.0001). After oral administration of low-dose and high-dose SBGP1, the protein level in BALF significantly decreased (*p* < 0.0001). Compared with the SBGP1-L group, the SBGP1-H group had a 25.26% decrease in protein levels in BALF.

The dynamic balance between pro-inflammatory cytokines and anti-inflammatory cytokines is crucial for the occurrence and development of the inflammatory response. To explore the effects of SBGP1 on the inflammatory response, the levels of inflammatory cytokines were quantified. The expression of pro-inflammatory cytokines such as TNF-*α*, IL-6, and IL-1*β* was significantly elevated in the M group compared with the C group, and these levels were reduced by SBGP1 treatment, showing a dose-dependent relationship (Figure 3E–G, *p* < 0.01). The M group presented significantly lower levels of anti-inflammatory cytokines, including IL-4 and IL-10, compared with the C group, which were increased evidently after the oral administration of SBGP1 (Figure 3H,I, *p* < 0.01). Low-dose SBGP1 and high-dose SBGP1 intake treatment increased IL-4 levels by 28.83% and 44.68%, respectively. Concurrently, low-dose SBGP1 and high-dose SBGP1 intake treatment increased IL-10 levels by 162.95% and 220.33%, respectively. These results indicated that SBGP1 intake treatment could significantly alleviate the inflammatory response in ALI mice.

### 3.3. SBGP1 Alleviated Oxidative Stress and Tissue Damage in ALI Mice

**Figure 4:**
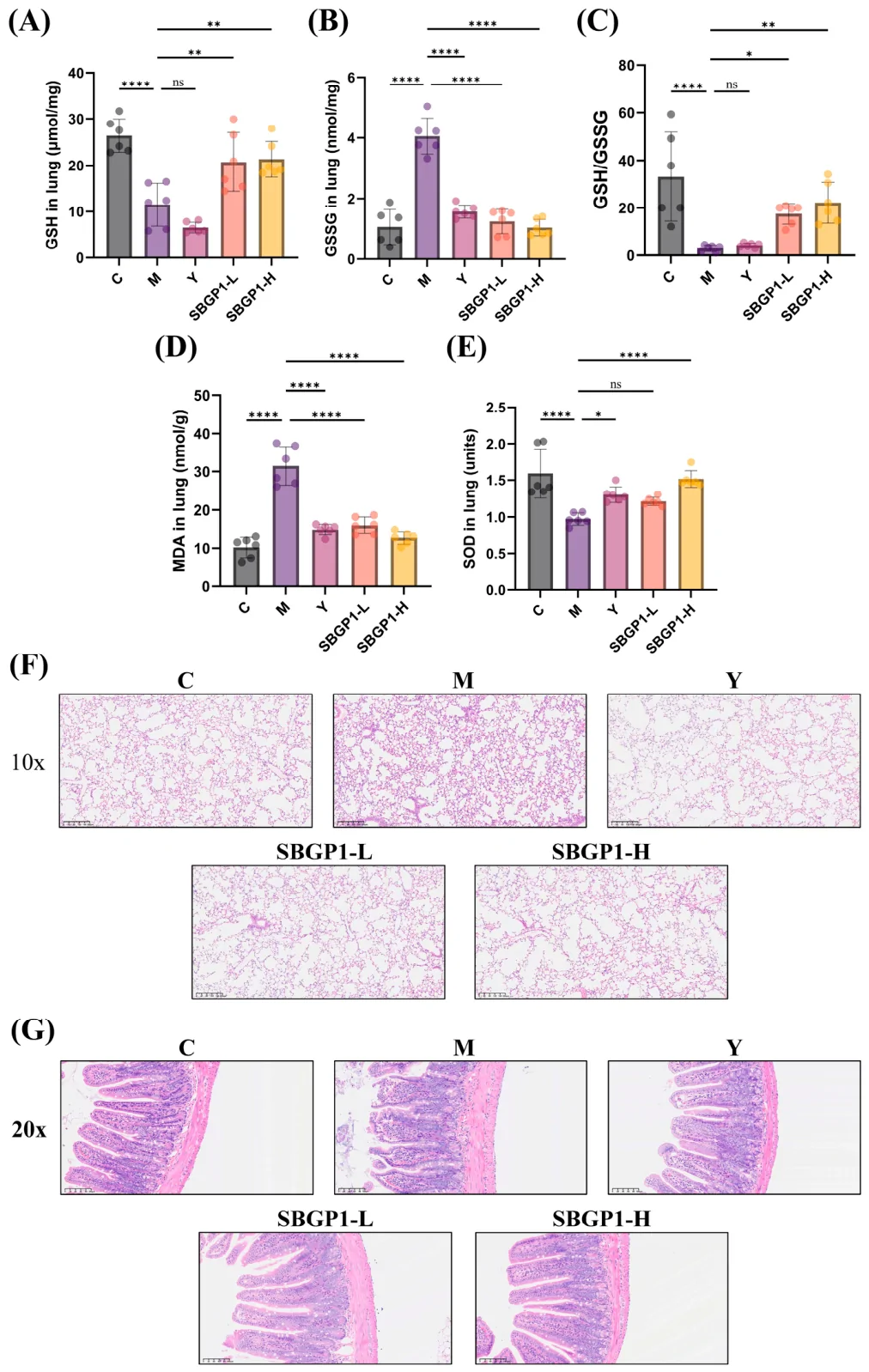
The relative levels of GSH (**A**), GSSG (**B**), GSH/GSSG (**C**), MDA (**D**), and SOD (**E**); H&E staining of lung tissues (**F**) and intestinal tissues (**G**). All data represent *n* = 6 independent mice per group. * *p* < 0.05, ** *p* < 0.01, *** *p* < 0.001, and **** *p* < 0.0001.

The effects of SBGP1 on oxidative stress were evaluated. As depicted in Figure 4A–C, compared with the C group, LPS stimulation significantly down-regulated the GSH level (*p* < 0.0001) and increased the GSSG level (*p* < 0.0001), as well as decreased the GSH/GSSG ratio (*p* < 0.0001). Compared with the M group, low-dose and high-dose SBGP1 intervention presented a significant therapeutic effect on these oxidative stress-related indexes such as GSH, GSSG and GSH/GSSG (*p* < 0.05). Moreover, the MDA level significantly increased in the M group compared with the C group, which was reduced by low-dose and high-dose SBGP1 intake treatment, with reductions of 49.03% and 59.59%, respectively (Figure 4D, *p* < 0.0001). Compared with the C group, SOD level significantly decreased in the M group (Figure 4E, *p* < 0.0001). Notably, oral administration of high-dose SBGP1 reversed the alterations (*p* < 0.0001), while low-dose treatment did not significantly improve SOD activity (*p* > 0.05). The data imply that SBGP1 treatment significantly alleviated oxidative stress in ALI mice.

Pathological analysis was performed to assess the protective effects of SBGP1 on ALI. As shown in Figure 4F, the C group presented normal morphology of lung tissues and no inflammatory cell infiltration. LPS stimulation resulted in widened alveolar septa, collapsed alveoli, disrupted alveolar structures, and significant neutrophil infiltration. After oral administration of SBGP1, these changes were evidently alleviated. Furthermore, SBGP1 also reversed LPS-induced shedding of small intestinal villi in intestinal tissue (Figure 4G).

### 3.4. SBGP1 Alleviated ALI Through Rap1 Signaling Pathway

**Figure 5:**
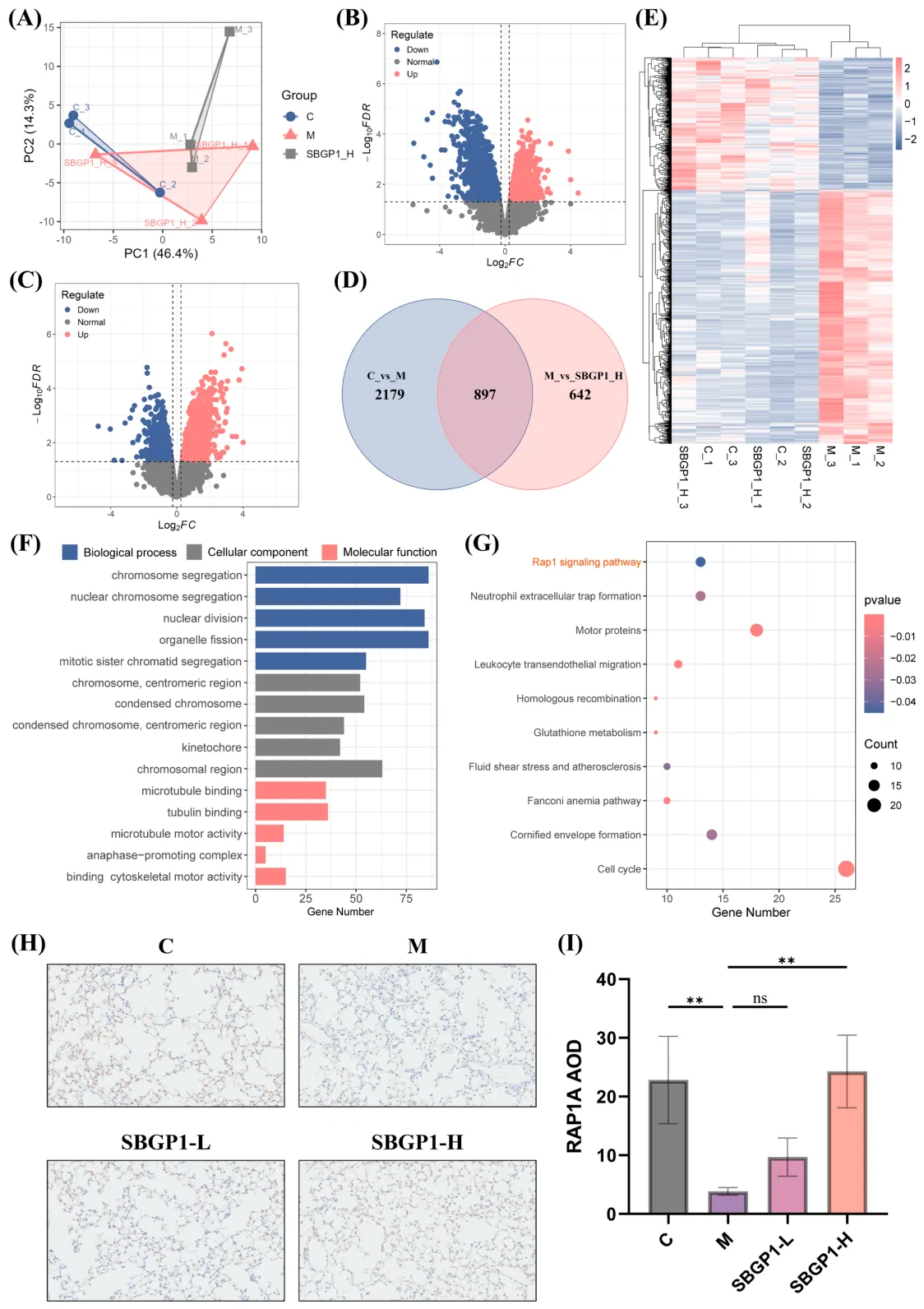
PCA score plot (**A**); DEGs screened between C and M groups (**B**); DEGs screened between M and SBGP1-H groups (**C**); Venn plot (**D**); clustering heatmap of DEGs (**E**); GO (**F**) and KEGG (**G**) enrichment analysis; immunohistochemical staining (**H**) and AOD of RAP1A expression in mouse lung tissue (**I**). All data represent *n* = 3 independent mice per group. * *p* < 0.05, ** *p* < 0.01, *** *p* < 0.001, and **** *p* < 0.0001.

Transcriptomic analysis was conducted to further elucidate the mechanism by which SBGP1 alleviates ALI. PCA score plot showed a separation among C, M, and SBGP1-H groups (Figure 5A). Notably, the SBGP1-H group was located between the C and M groups, indicating that SBGP1 intake treatment reversed LPS-induced changes in gene expression. Differential expression genes (DEGs) were screened based on criteria of |Log_2_FC| ≥ 0.25 and adjusted *p* < 0.05. A total of 3076 DEGs were identified between the C and M groups, including 1770 up-regulated and 1306 down-regulated (Figure 5B). In the comparison between the M and SBGP1-H groups, a total of 1539 DEGs were screened, including 946 up-regulated and 593 down-regulated (Figure 5C). The Venn diagram showed that SBGP1 predominantly alleviated ALI by regulating the expression of 897 DEGs (Figure 5D). The clustering heatmap was built to present the relative levels of these DEGs (Figure 5E). The expression levels of the C group and SBGP1-H group were similar and clustered together, indicating that SBGP1 mainly alleviated ALI through these DEGs. Subsequently, GO and KEGG enrichment analysis of DEGs was conducted. As shown in Figure 5F, GO enrichment analysis mainly involved three categories, including biological process, cellular component, and molecular function. Meanwhile, DEGs were mainly enriched in biological processes such as chromosome segregation, nuclear division, and organelle fission; cellular components such as the chromosomal region, condensed chromosome, and chromosome, centromeric region; and molecular functions such as tubulin binding, microtubule binding, and microtubule motor activity. KEGG pathway enrichment analysis showed that DEGs were primarily enriched in pathways such as the Rap1 signaling pathway, cell cycle, cornified envelope formation, motor proteins, and leukocyte transendothelial migration (Figure 5G). These results indicate that the Rap1 signaling pathway was one of the pathways through which SBGP1 alleviated ALI.

Immunohistochemical analysis was performed to determine the levels of RAP1A in lung tissue of mice (Figure 5H). Compared with the C group, LPS stimulation significantly down-regulated the levels of RAP1A, while high-dose SBGP1 intake treatment evidently increased its expression, indicating that SBGP1 activated the Rap1 signaling pathway, which may contribute to alleviating ALI (Figure 5I, *p* < 0.01).

### 3.5. SBGP1 Modulated Gut Microbiota and SCFA Contents of ALI Mice

Gut microbiota has become an important target for various diseases. The effects of gut microbiota in SBGP1 alleviating ALI were evaluated using pseudo-germ-free mice (Figure S3A). As depicted in Figure S3B, the body weight of all groups of mice increased with the prolongation of days, indicating that fecal microbiota derived from SBGP1 group mice did not produce significant side effects. Compared with the C group, the M group showed increased lung index, while the SBGP1 group reversed this change (Figure S3C, *p* < 0.05). However, administration of high-dose SBGP1 to pseudo-germ-free mice did not significantly reduce lung index (*p* > 0.05). As shown in Figure S3D, compared with the M group, protein concentration in BALF of GM-K and FMT-SBGP1 groups decreased by 34.48% and 65.48%, respectively (*p* < 0.05). Notably, fecal microbiota derived from the SBGP1 group significantly alleviated inflammatory response and oxidative stress, as evidenced by decreased levels of TNF-*α*, IL-6, IL-1*β*, GSSG and MDA, and increased levels of IL-4, IL-10, GSH, GSH/GSSG ratio, and SOD (Figure S3E–N, *p* < 0.01). However, administration of high-dose SBGP1 to pseudo-germ-free mice did not significantly reverse these alterations (*p* > 0.05). These results suggested that gut microbiota are important targets for SBGP1 in alleviating ALI.

**Figure 6:**
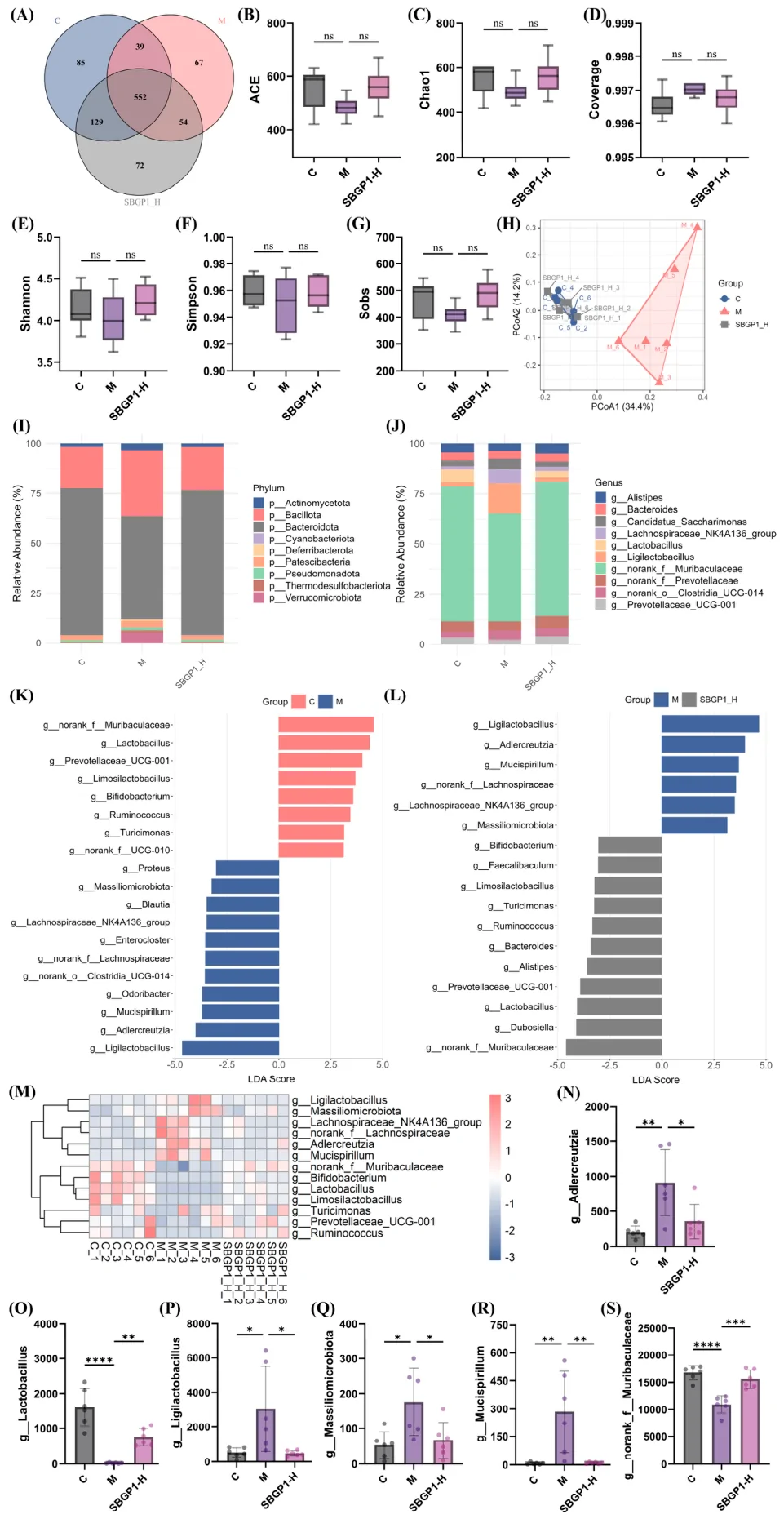
Venn plot (**A**); Alpha diversity: ACE (**B**), Chao1 (**C**), Coverage (**D**), Shannon (**E**), Simpson (**F**), Sobs (**G**); PCoA score plot (**H**); Changes in relative abundance of gut microbiota at the phylum (**I**) and genus (**J**) levels; Differential genera between C and M groups (**K**); Differential genera between M and SBGP1 groups (**L**); The clustering heatmap of common differential genera (**M**); The relative abundance of differential genera: *Adlercreutzia* (**N**), *Lactobacillus* (**O**), *Ligilactobacillus* (**P**), *Massiliomicrobiota* (**Q**), *Mucispirillum* (**R**), *norank_f__Muribaculaceae* (**S**). All data represent *n* = 6 independent mice per group. * *p* < 0.05, ** *p* < 0.01, *** *p* < 0.001, and **** *p* < 0.0001.

Subsequently, gut microbiota analysis was conducted. As illustrated in Figure 6A, a total of 552 OTUs were identified based on the similarity of the sequences. Then, alpha diversity was assessed, and the results showed that indices, including ACE, Chao1, Coverage, Shannon, Simpson and Sobs, did not significantly change among C, M, and SBGP1 groups, suggesting no significant change in species diversity (Figure 6B–G). Beta diversity was performed to evaluate the differences in the composition and structure of microbial communities. The PCoA score plot showed that the M group was clearly distinguished from the C and SBGP1 groups, while the C and SBGP1 groups clustered together, implying that SBGP1 intervention restored the disorder of microbial communities (Figure 6H). Subsequently, the changes in relative abundance of gut microbiota among these groups at the phylum and genus levels were explored. At the phylum level, compared with the C group, the M group presented higher relative abundance of Actinomycetota, Bacillota and Verrucomicrobiota, and lower relative abundance of Bacteroidota (Figure 6I). SBGP1 intake treatment reversed these changes. At the genus level, LPS stimulation increased the relative abundance of *Ligilactobacillus*, *Lachnospiraceae_NK4A136_group*, and *Candidatus Saccharimonas*, and decreased the relative abundance of *Lactobacillus*, *norank_f__Muribaculaceae*, and *Prevotellaceae_UCG−001* (Figure 6J). SBGP1 restored the abnormal relative abundance of these genera.

LEfSe analysis was performed to identify differential genera. As shown in Figure 6K, a total of 19 genera were identified between the C and M groups. Among them, the C group showed higher relative abundance of *norank_f__Muribaculaceae*, *Lactobacillus*, and *Limosilactobacillus*, while the M group presented higher relative abundance of *Ligilactobacillus*, *Adlercreutzia*, and *Mucispirillum*. In the comparison of the M and SBGP1-H groups, genera such as *norank_f__Muribaculaceae*, *Dubosiella*, and *Lactobacillus*, were dominant genera in the SBGP1-H group (Figure 6L). After integrating these screening results, 13 differential genera were identified, and their relative abundances were presented in Figure 6M. Compared with the C group, LPS stimulation significantly up-regulated the relative abundance of genera such as *Adlercreutzia*, *Ligilactobacillus*, *Massiliomicrobiota*, and *Mucispirillum* (Figure 6N, 6P-R, *p* < 0.05), and down-regulated the relative abundance of genera such as *Lactobacillus* and *norank_f__Muribaculaceae* (Figure 6O,S, *p* < 0.0001). SBGP1 intake treatment significantly reversed these changes (*p* < 0.05). These results suggested that SBGP1 could alleviate ALI by restoring gut microbiota dysbiosis, mainly by increasing the abundance of genera such as *Lactobacillus* and *norank_f__Muribaculaceae*. Functional prediction analysis suggested that differential genera primarily involved multiple pathways, including galactose metabolism, cAMP signaling pathway, carbohydrate digestion and absorption, glycolysis/gluconeogenesis, and estrogen signaling pathway (Figure S4). Notably, at the species level, there was no *norank_f__Muribaculaceae* strain suitable for microbiota transplantation. Therefore, a key bacterial species (*Lactobacillus johnsonii*) was selected to verify its effectiveness against ALI. As depicted in Figure S5, compared with the C group, LPS intervention significantly decreased the relative abundance of *Lactobacillus johnsonii* (*p* < 0.0001), while SBGP1 significantly increased its abundance (*p* < 0.01).

### 3.6. Lactobacillus Johnsonii Inhibited Excessive Activation of Inflammatory Response and Oxidative Stress, and Secreted Propionic Acid

**Figure 7:**
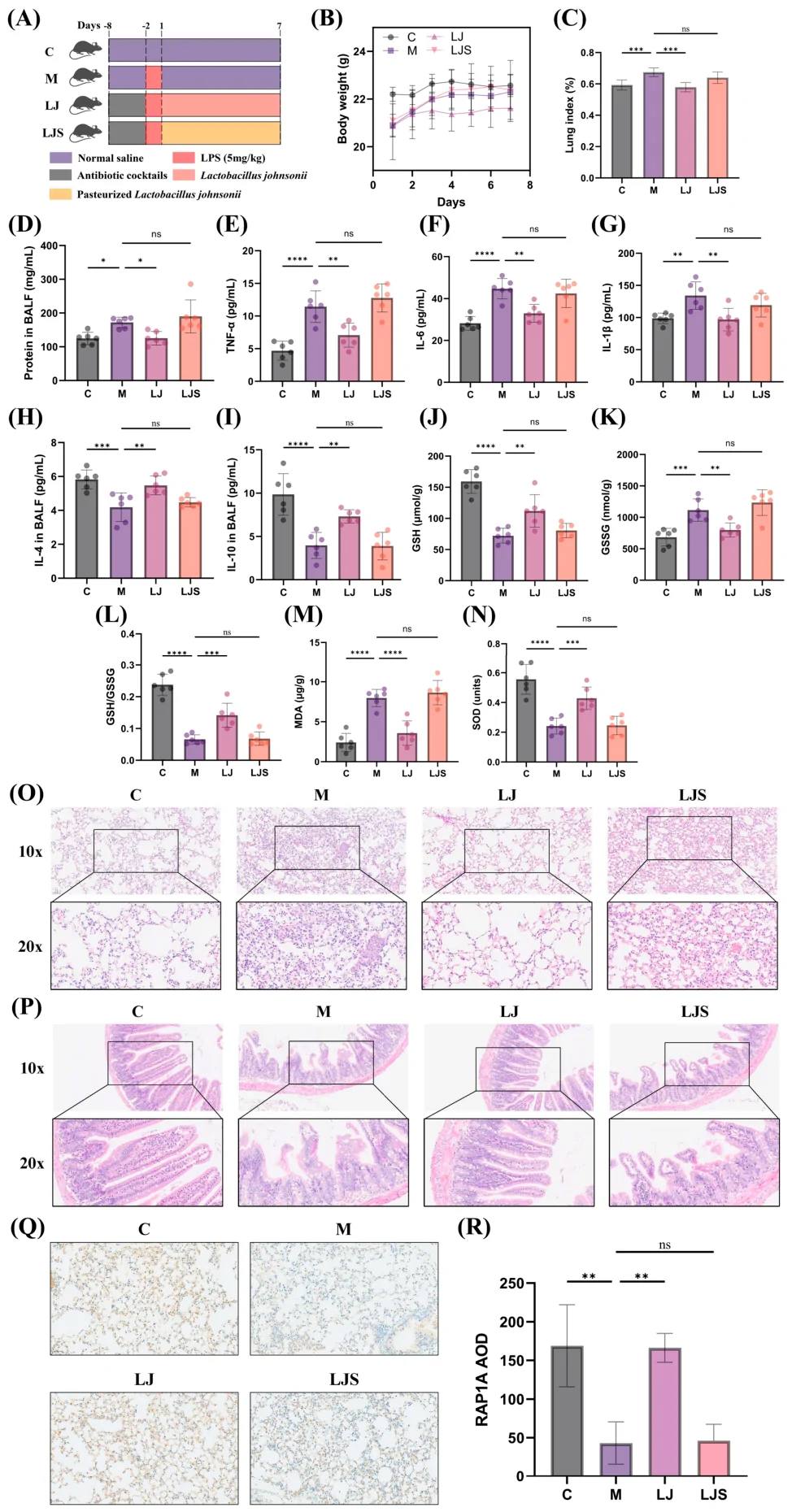
Experimental design of *Lactobacillus johnsonii* against ALI (**A**); Changes in body weight of ALI mice (**B**); Changes in lung index (**C**); The relative levels of protein (**D**), TNF-*α* (**E**), IL-6 (**F**), IL-1*β* (**G**), IL-4 (**H**), and IL-10 (**I**) in BALF; The relative levels of GSH (**J**), GSSG (**K**), GSH/GSSG (**L**), MDA (**M**), and SOD (**N**); H&E staining of lung tissues (**O**) and intestinal tissues (**P**); Immunohistochemical staining (**Q**) and AOD of RAP1A expression in mice lung tissue (**R**). All data represent *n* = 6 independent mice per group. * *p* < 0.05, ** *p* < 0.01, *** *p* < 0.001, and **** *p* < 0.0001.

*Lactobacillus johnsonii* is a commensal bacterium with numerous health benefits, including restoring the intestinal barrier, immune regulation, and anti-inflammatory effects [30]. To further assess the protective effect of *Lactobacillus johnsonii* on ALI, viable *Lactobacillus johnsonii* and pasteurized *Lactobacillus johnsonii* were transplanted into the intestines of ALI mice (Figure 7A). As depicted in Figure 7B, the body weight of all groups of mice increased with the prolongation of days, indicating that viable *Lactobacillus johnsonii* and pasteurized *Lactobacillus johnsonii* did not produce significant side effects. Compared with the M group, viable *Lactobacillus johnsonii* significantly decreased lung index and protein concentration in BALF (*p* < 0.05), while pasteurized *Lactobacillus johnsonii* did not reverse these changes (Figure 7C,D, *p* > 0.05). Similarly, viable *Lactobacillus johnsonii* significantly restored the balance of inflammatory response, as evidenced by decreased levels of TNF-*α*, IL-1*β*, and IL-6, and increased levels of IL-4 and IL-10 (Figure 7E–I, *p* < 0.05). Furthermore, viable *Lactobacillus johnsonii* significantly up-regulated the decreased levels of GSH, GSH/GSSG ratio, and SOD, and down-regulated the elevated levels of GSSG and MDA induced by LPS stimulation, ultimately restoring the balance of oxidative stress (Figure 7J–N, *p* < 0.05). However, pasteurized *Lactobacillus johnsonii* did not inhibit excessive activation of the inflammatory response and oxidative stress (*p* > 0.05). Furthermore, viable *Lactobacillus johnsonii* alleviated LPS-induced lung and intestinal tissue injury, whereas pasteurized *Lactobacillus johnsonii* did not have this effect (Figure 7O,P). Similarly, the expression of RAP1A in lung tissue was evaluated by immunohistochemical analysis (Figure 7Q). Compared with the LJS group, the level of RAP1A in the LJ group increased by 72.26%, and the difference was statistically significant (Figure 7R, *p* < 0.01). These results indicated that the observed protective effect of *Lactobacillus johnsonii* predominantly depended on microbial metabolites rather than the bacterial components.

**Figure 8:**
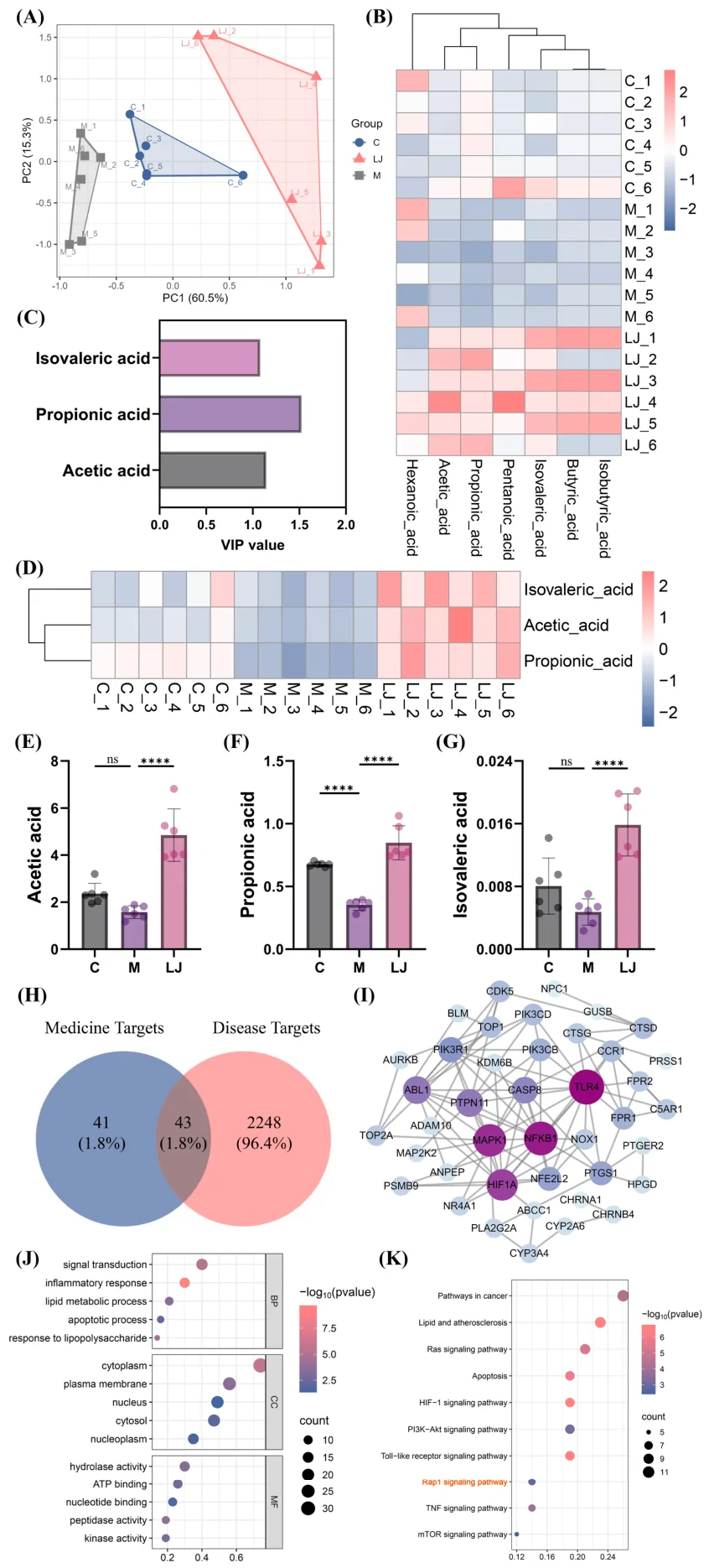
PCA score plot (**A**); Clustering heatmap of SCFAs (**B**); Differential bacterial metabolites screened by PLS-DA (**C**); Clustering heatmap of differential SCFAs (**D**); The relative content of SCFAs: Acetic acid (**E**), Propionic acid (**F**), Isovaleric acid (**G**); Venn plot (**H**); PPI topological network (**I**); GO (**J**) and KEGG (**K**) pathway enrichment analysis. All data represent *n* = 6 independent mice per group. * *p* < 0.05, ** *p* < 0.01, *** *p* < 0.001, and ****p < 0.0001.

To screen key mediators for *Lactobacillus johnsonii* against ALI, targeted metabolomics analysis was conducted. As shown in Figure 8A, the C, M, and LJ groups were clearly distinguished, suggesting differences in their SCFA profiles. Compared with the C group, LPS stimulation decreased SCFA levels, while *Lactobacillus johnsonii* reversed these changes (Figure 8B). Subsequently, partial least squares-discriminant analysis (PLS-DA) was conducted to identify differential SCFAs. As shown in Figure 8C, a total of three differential SCFAs with VIP > 1 were selected, including acetic acid, propionic acid and isovaleric acid. The clustering heatmap of these differential SCFAs showed that higher levels of these SCFAs were found in the C and LJ groups than in the M group. Notably, among these three SCFAs, only propionic acid showed significant differences between the C and M groups (Figure 8E–G, *p* < 0.0001). Therefore, propionic acid was regarded as a key mediator for *Lactobacillus johnsonii* in alleviating ALI.

Network pharmacology was used to predict the mechanism of propionic acid in alleviating ALI. Based on the SuperPRED platform and the GeneCards database, a total of 84 medicine targets and 2291 disease targets were collected, respectively. As shown in Figure 8H, 43 potential targets were identified by the Venny platform. Subsequently, the PPI topological network suggested that these potential targets exhibited complex interactions, and targets such as TLR4, MAPK, and NFKB1 could regulate other targets, thereby exerting pharmacological effects (Figure 8I). Additionally, GO enrichment analysis suggested that potential targets were mainly enriched in biological processes such as signal transduction, inflammatory response, and lipid metabolic process; cellular components such as cytoplasm, plasma membrane and nucleus; and molecular functions such as hydrolase activity, ATP binding and nucleotide binding (Figure 8J). KEGG pathway enrichment analysis showed that propionic acid could alleviate ALI by regulating pathways such as the HIF-1 signaling pathway, PI3K-Akt signaling pathway, Toll-like receptor signaling pathway, Rap1 signaling pathway, and TNF signaling pathway (Figure 8K). Notably, transcriptomic analysis also showed the important role of the Rap1 signaling pathway for SBGP1 in alleviating ALI, which demonstrated the importance of the Rap1 signaling pathway in the treatment of ALI with SBGP1.

### 3.7. Propionic Acid Restored Excessive Activation of Inflammatory Response and Oxidative Stress in ALI Mice

**Figure 9:**
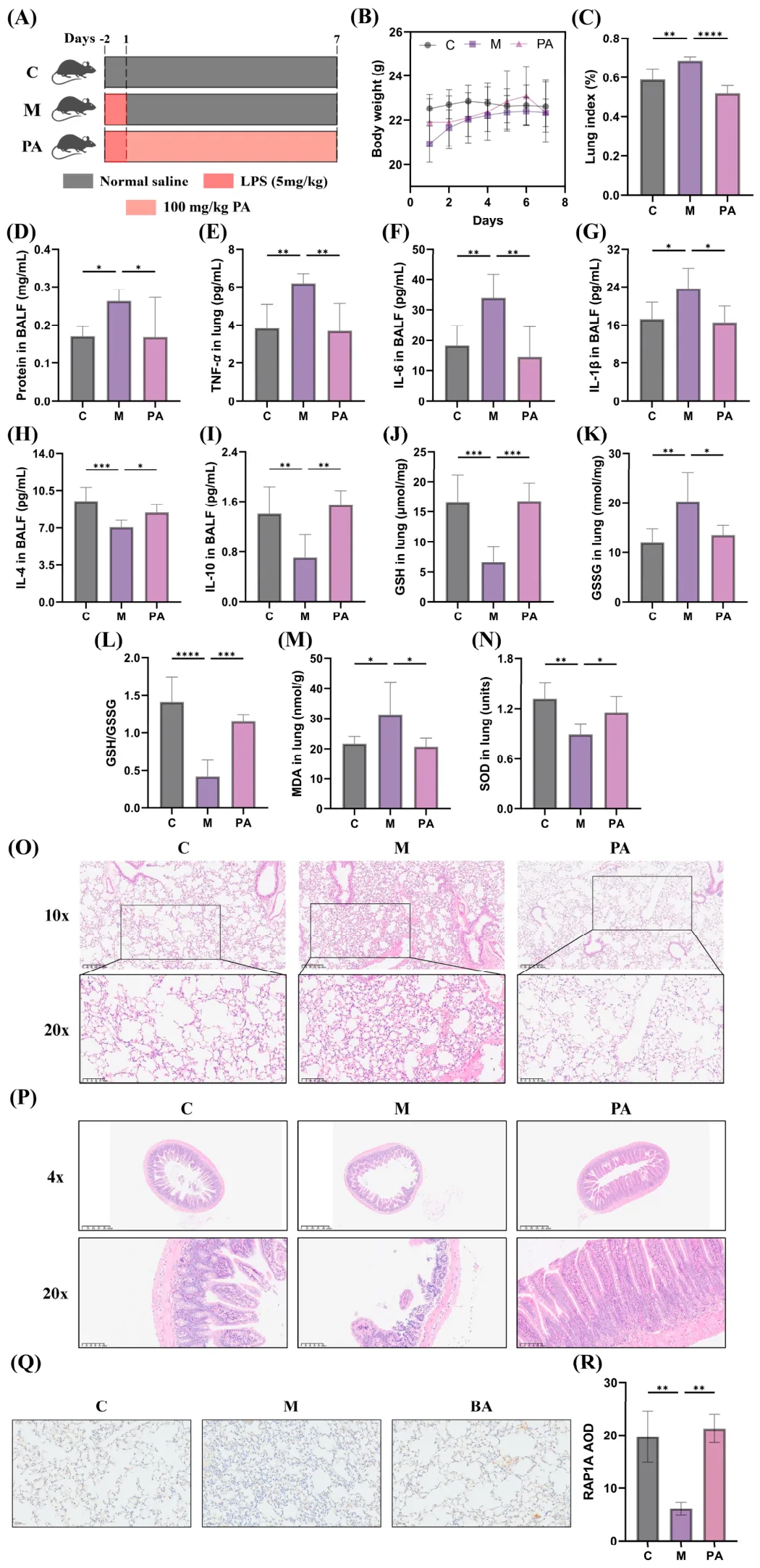
Experimental design of propionic acid against ALI (**A**); Changes in body weight of ALI mice (**B**); Changes in lung index (**C**); The relative levels of protein (**D**), TNF-*α* (**E**), IL-6 (**F**), IL-1*β* (**G**), IL-4 (**H**), and IL-10 (**I**) in BALF; The relative levels of GSH (**J**), GSSG (**K**), GSH/GSSG (**L**), MDA (**M**), and SOD (**N**); H&E staining of lung tissues (**O**) and intestinal tissues (**P**); Immunohistochemical staining (**Q**) and AOD of RAP1A expression in mice lung tissue (**R**). All data represent *n* = 6 independent mice per group. * *p* < 0.05, ** *p* < 0.01, *** *p* < 0.001, and **** *p* < 0.0001.

Administration of propionic acid was performed to evaluate its effectiveness against ALI (Figure 9A). As depicted in Figure 9B, propionic acid intervention did not cause any abnormal changes in body weight. Compared with the C group, LPS stimulation significantly increased indices such as the lung index and protein concentration in BALF, while propionic acid intake treatment reversed these changes (Figure 9C,D, *p* < 0.05). Meanwhile, propionic acid significantly inhibited the excessive activation of the inflammatory response, as evidenced by decreased levels of TNF-*α*, IL-1*β*, and IL-6, and increased levels of IL-4 and IL-10 (Figure 9E–I, *p* < 0.05). As depicted in Figure 9J–N, propionic acid evidently up-regulated the levels of GSH, GSH/GSSG ratio, and SOD (*p* < 0.05), as well as down-regulated the levels of GSSG and MDA (*p* < 0.05), indicating propionic acid could restore the imbalance of oxidative stress. As shown in Figure 9O, compared with the C group, LPS stimulation caused widened alveolar septa, collapsed alveoli, disrupted alveolar structures, and significant neutrophil infiltration, while propionic acid intake treatment improved the lung injury. Additionally, propionic acid also reversed some abnormal changes such as shedding of small intestinal villi and reduction in the number of goblet cells (Figure 9P). Notably, propionic acid significantly reversed the decreased levels of RAP1A caused by LPS stimulation (Figure 9Q,R, *p* < 0.01).

## 4. Discussion

SBG polysaccharides have attracted increasing academic attention due to their potential anti-inflammatory effects, regulation of intestinal homeostasis, and immunomodulation properties [12,15,16]. In this study, a novel SBG polysaccharide fraction (SBGP1) was isolated and purified from SBG. SBGP1 was a pectic polysaccharide characterized by high proportions of Gal*p*A and methyl-esterified Gal*p*A, indicating an unsubstituted homogalacturonan (HG) structure. Its degree of methylation (DM) value was 39.46%, suggesting that SBGP1 was a low-methoxyl pectin (LMP) [31]. Methylation analysis revealed that SBGP1 contained 2.06% of 1,2,4-linked-Rha*p*, along with arabinose-related glycosidic residues, implying the presence of a small amount of rhamnogalacturonan-I (RG-I). Although methylation analysis provided evidence for the existence of HG and RG-I domains, NMR analysis limited the signal assignment for the RG-I backbone. As a prebiotic, pectin has a retention rate of over 95% in the large intestine and a low blood entry rate [32]. Meanwhile, low-molecular weight pectin is more easily degraded by gut microbiota, which can be attributed to its small size, simplified structure, and low spatial hindrance [33]. Therefore, the average *M*w of SBGP1 was 40,764.6 Da, which promoted its utilization by gut microbiota and the production of SCFAs. Notably, pectin rich in HG domains has been shown to inhibit the excessive production of pro-inflammatory mediators such as TNF-*α*, IL-1*β*, and IL-6, while restoring the balance of anti-inflammatory cytokines including IL-4 and IL-10, and alleviating acute inflammation by inhibiting the NF-κB and p38/JNK MAPK signaling pathways. In contrast, RG-I pectin mainly exerts anti-inflammatory effects by binding to galectin-3, which is more closely related to chronic inflammation [34]. In the present study, SBGP1 is predominantly characterized by HG domains, which is consistent with its alleviating effects on ALI, suggesting that the HG domain plays a major role in the protective activity against ALI, while the role of RG-I domains is relatively small.

The pathogenesis of ALI is closely associated with neutrophil recruitment and accumulation in the lung, where LPS activates these neutrophils to trigger inflammatory responses and oxidative stress, ultimately leading to edema and lung injury [3]. The levels of TNF-*α*, IL-1*β*, and IL-6 in BALF were widely recognized as indicators for assessing the severity of the inflammatory response [35]. After lung tissue was damaged, macrophages secreted various pro-inflammatory cytokines, including TNF-*α*, IL-1*β*, and IL-6 [36]. The inflammatory response was also accompanied by the reduced secretion of anti-inflammatory cytokines such as IL-4 and IL-10. Additionally, TNF-*α* could induce oxidative stress in tissues. Our findings showed that LPS-induced ALI mice exhibited significantly elevated levels of TNF-*α*, IL-1*β*, and IL-6 as well as decreased levels of IL-4 and IL-10, indicating the occurrence of an inflammatory response. Meanwhile, oxidative stress indices were also higher in ALI mice, as evidenced by increased levels of GSSG and MDA, and decreased levels of GSH and SOD. Notably, SBGP1 intake treatment effectively reversed these changes, indicating SBGP1 could alleviate ALI by restoring the balance of inflammatory response and oxidative stress. In addition, although dexamethasone also showed therapeutic effects on ALI, it induced weight loss in ALI mice, which was consistent with the side effects of glucocorticoids, including protein catabolism, insulin resistance, and metabolic disorders. These adverse effects limited the long-term use of glucocorticoids and may even induce secondary complications. Therefore, SBGP1 has significant potential for treating ALI.

Transcriptomic analysis was conducted to reveal the underlying mechanism of SBGP1 against ALI. Our findings suggested that the treatment of ALI with SBGP1 may be mainly related to the activation of the Rap1 signaling pathway. A previous study suggested that Rap1 was closely related to ALI and intestinal mucosal injury [37]. Moreover, the Rap1 signaling pathway could regulate macrophage polarization, which included activated M1 and M2 macrophages [37,38]. Activated M1 macrophages induced an inflammatory response by secreting pro-inflammatory cytokines such as IL-1*β*, TNF-*α* and IL-6, while activated M2 macrophages alleviated the inflammatory response by secreting anti-inflammatory cytokines such as IL-4 and IL-10 [39,40]. In the present study, LPS stimulation significantly decreased the expression of Rap1A compared with the C group, while oral administration of SBGP1 restored the altered change. Therefore, SBGP1 may alleviate ALI by activating the Rap1 signaling pathway, further restoring the dynamic balance of M1 and M2 macrophages, ultimately decreasing the levels of pro-inflammatory cytokines such as IL-1*β*, TNF-*α* and IL-6, as well as increasing the levels of anti-inflammatory cytokines such as IL-4 and IL-10.

The gut microbiota has emerged as an important therapeutic target for ALI due to its capacity to preserve intestinal homeostatic balance, modulate host immune responses, and regulate the homeostatic state and pathological progression of the distal lung [41]. This is mainly because metabolites of gut microbiota can be transported through the bloodstream, target the lungs, and regulate immune responses and inflammation by altering the lung immune microenvironment, thereby driving or controlling the progression of acute inflammation [41]. In the present study, our findings showed that at the level of phylum, SBGP1 decreased the relative abundance of Actinomycetota, Bacillota and Verrucomicrobiota and increased the relative abundance of Bacteroidota. Interestingly, Bacteroidota could encode many carbohydrate-degrading enzymes [42]. Elevated abundance of Bacteroidota may be attributed to a better growing environment induced by SBGP1. At the genera level, the decrease in relative abundance of *norank_f__Muribaculaceae* has been shown to potentially induce the disorder in inflammatory response, oxidative stress, and SCFAs production in colitis mice [43,44]. As an important probiotic, a decrease in the relative abundance of *Lactobacillus* may lead to damage to the intestinal mucosal barrier, decreased host immune function, and decreased gut microbiota metabolites [45,46]. Additionally, SBGP1 significantly increased the relative abundance of genera such as *Lactobacillus* and *norank_f__Muribaculaceae*, thereby reversing these abnormal alternations. Functional prediction analysis suggested that differential genera alleviate ALI by regulating multiple pathways. Firstly, these differential bacterial genera can regulate carbohydrate metabolism. Compared with the C group, the abundance of galactose metabolism and carbohydrate digestion and absorption in the M group decreased, indicating that carbohydrate metabolism in the M group was inhibited to a certain extent. In contrast, the carbohydrate metabolism of the SBGP1-H group returned to normal. Secondly, the relative abundance of the cAMP signaling pathway in the C and SBGP1-H groups was higher than that in the M group, indicating that the differential bacterial genera in the C and SBGP1-H groups induced upregulation of cAMP levels and promoted activation of the cascade reaction (cAMP-RAPGEF3-Rap1). Thirdly, the abundance of other pathways such as glycolysis/gluconeogenesis and the estrogen signaling pathway in the C and SBGP1-H groups was also higher than that in the M group. Among them, glycolysis is the process by which glucose is broken down into pyruvate in the cytoplasm, and the enhancement of glycolysis has been shown to promote inflammation and oxidative stress [47]. Interestingly, compared with the M group, the SBGP1-H group showed a higher abundance of glycolysis, indicating that it may be one of the potential pathways for SBGP1 to alleviate ALI. As a key steroid hormone, the decrease in estrogen levels exacerbates LPS-induced mitochondrial damage, cell apoptosis, and lung injury [48]. Compared with the M group, the relative abundance of the estrogen signaling pathway in the SBGP1-H group was higher, indicating that SBGP1 could mediate gut microbiota, indirectly upregulate the estrogen signaling pathway, and alleviate ALI. Further analysis showed that *Lactobacillus johnsonii* was identified as a key bacterial species for SBGP1 against ALI. Mechanistically, this protection may involve a complex regulatory network. On the one hand, *Lactobacillus johnsonii* has been shown to inhibit lung inflammation through the Oxindole AhR-CXCL13 axis [49]. On the other hand, *Lactobacillus johnsonii* has also been demonstrated to increase propionic acid in the intestine, which is consistent with the findings of this study [50]. Interestingly, propionic acid has been demonstrated to alleviate ALI by restoring inflammatory responses and reducing oxidative stress [30], and our study further reveals its positive impact on the activation of the Rap1 signaling pathway. Collectively, these findings suggest that SBGP1 may alleviate ALI by enriching *Lactobacillus johnsonii*, which in turn elevates propionic acid levels and promotes Rap1 signaling activation. The significantly decreased abundance of *Lactobacillus johnsonii* observed in ALI patients further supports its beneficial role in mitigating ALI [49].

At present, to the best of our knowledge, the anti-inflammatory effect of SBG polysaccharides mainly relies on multiple signaling pathways, including NF-κB, MLCK/MLC, Notch, JAK2/STAT3, and the IL-23/IL-17 axis [51]. In this study, we demonstrated that a homogeneous acidic polysaccharide (SBGP1) with a unique HG-rich structure may alleviate ALI through the *Lactobacillus johnsonii*-propionic acid-Rap1 pathway. Specifically, SBGP1 increased the relative abundance of *Lactobacillus johnsonii* and induced the production of propionic acid, which may enter the circulation and activate the Rap1 signaling pathway in the lung, thereby promoting M2 macrophage polarization to attenuate excessive activation of the inflammatory response.

However, several limitations of this study should be acknowledged. Firstly, the network pharmacology analysis of PA currently only remains at the prediction level. Further verification on the regulatory effects of PA on other signaling pathways needs to be carried out in the future. Secondly, NMR analysis has only assigned the chemical shifts of 7 major glycosidic residues, and the stepwise acid hydrolysis method can be adopted in subsequent studies to further characterize its complete structure. Thirdly, the spatial structure of SBGP1 has not yet been elucidated, and molecular dynamics simulations should be employed in the future to further reveal its conformation. Fourthly, although the Rap1 signaling pathway has been shown to be potentially associated with SBGP1 in alleviating ALI, this study only validated the expression of RAP1A, and future research will further verify changes in the levels of other related proteins. Fifthly, this study only speculated that SBGP1 may regulate specific polarization of macrophages, and its regulatory effect should be further validated by flow cytometry in the future.

## 5. Conclusions

In summary, a homogeneous acidic polysaccharide, SBGP1, was isolated and purified from *Scutellaria baicalensis* Georgi, and its structural characterization and impact on ALI were investigated. Structural analysis suggested that SBGP1 was a homogeneous polysaccharide with an average *M*w of 40764.6 Da and mainly consisted of GalA (53.14%), Ara (26.99%), Gal (7.75%), Glc (5.95%) and Rha (6.17%). The main chain of SBGP1 was composed of →4)-*α*-Gal*p*A-6-OMe-(1→ and →4)-*α*-Gal*p*A-(1→. Pharmacological studies showed that SBGP1 alleviated inflammatory response and oxidative stress, as evidenced by decreased levels of TNF-*α*, IL-1*β*, IL-6, GSSG and MDA, and increased levels of IL-4, IL-10, GSH and SOD. Moreover, SBGP1 was associated with activation of the Rap1 signaling pathway. Concurrently, SBGP1 alleviated ALI by increasing the relative abundance of *Lactobacillus* and *norank_f__Muribaculaceae* and decreasing the relative abundance of *Adlercreutzia*, *Ligilactobacillus*, *Massiliomicrobiota* and *Mucispirillum*. FMT treatment with SBGP1-derived microbiome evidently improved inflammatory response and oxidative stress in ALI mice. More importantly, SBGP1 up-regulated the relative abundance of *Lactobacillus johnsonii* as well as increased levels of propionic acid. Supplementation with *Lactobacillus johnsonii* and propionic acid also improved ALI. These positive effects provide evidence for the research and development of SBG acidic polysaccharides and offer new insights into potential therapeutic targets for ALI.

## Figures and Tables

**Table 1:** Methylation results of SBGP1.

Rt/min	Residues	Mass-to-Charge Ratio	Relative Molar Ratio (%)
10.86	T-linked-Ara*f*	71, 87, 102, 118, 129, 145, 161	8.06
14.45	T-linked-Gal*p*A	73, 89, 102, 118, 131, 147, 162, 163, 207	5.88
14.82	1,5-linked-Ara*f*	87, 102, 118, 129, 162, 189	3.62
16.4	1,3-linked-Gal*p*	87, 101, 102, 118, 129, 143, 162, 203	2.75
16.88	1,3,5-linked-Ara*f*	85, 99, 118, 127, 159, 201, 261	1.87
16.95	1,2,4-linked-Rha*p*	88, 101, 102, 118, 130, 143, 162, 203	2.06
17.10	1,4-linked-Gal*p*	87, 99, 102, 113, 118, 129, 131, 162, 173, 233	9.70
17.11	1,4-linked-Gal*p*A	87, 101, 102, 115, 118, 129, 133, 162, 175, 235	50.66
17.25	1,4-linked-Glc*p*	87, 99, 102, 113, 118, 129, 131, 162, 173, 233	2.71
17.40	1,2,5-linked-Ara*f*	87, 88, 129, 130, 189, 190	2.14
17.52	1,6-linked-Glc*p*	87, 99, 102, 118, 129, 162, 189, 233	4.51
18.64	1,3,4-linked-Gal*p*A	87, 118, 131, 145, 187, 205, 307	2.97
18.86	1,2,3,5-linked-Ara*f*	85, 86, 103, 115, 116, 128, 145, 146, 159, 188, 201, 218, 290	1.78
19.75	1,2,4-linked-Gal*p*A	88, 101, 115, 130, 175, 190, 235	1.28

**Table 2:** Assignment of sugar ring carbon/hydrogen signals of SBGP1.

Glycosidic Bond		H1/C1	H2/C2	H3/C3	H4/C4	H5/C5	H6/C6	-OMe
A	→4)-*α*-Gal*p*A-6-OMe-(1→ [27]	4.89/97.68	3.63/67.75	3.93/68.46	4.40/78.40	5.01/70.49	n.d./170.80	3.73/52.86
B	→4)-*α*-Gal*p*A-(1→ [27]	5.02/99.03	3.65/67.92	3.92/68.07	4.38/78.74	4.71/70.56	n.d./174.90	
C	→4)-*α*-Gal*p*-(1→ [28]	4.83/99.48	3.66/68.02	3.75/69.50	3.88/76.53	4.00/74.07	3.63/62.48	
D	*α*-Ara*f*-(1→ [28]	5.08/106.40	4.06/80.10	3.89/78.96	4.02/80.98	3.77/61.09	/	
E	*α*-Gal*p*A-(1→ [27]	5.07/100.93	3.89/68.07	4.03/69.87	4.22/71.20	4.71/70.67	n.d./174.90	
F	→6)-*α*-Glc*p*-(1→ [28]	4.90/97.68	3.50/71.37	3.63/73.38	3.43/69.50	3.83/70.15	3.61/65.58	
G	→5)-*α*-Ara*f*-(1→ [28]	5.11/106.92	4.05/81.10	3.87/76.68	4.00/80.91	3.65/67.92	/	

**Table 3:** Significant connections observed in the HMBC spectrum for anomeric proton/carbon of the sugar residues of SBGP1.

Sugar Residue	H1/C1	Observed Connectivity	
	*δ*H/*δC*	*δ*H/*δC*	Residue
A: →4)-*α*-Gal*p*A-6-OMe-(1→	4.89	78.74	B-C4
B: →4)-*α*-Gal*p*A-(1→	5.02	78.40/78.74/65.58	A-C4/B-C4/F-C6
C: →4)-*α*-Gal*p*-(1→	4.83	78.40	A-C4
D: *α*-Ara*f*-(1→	5.08	76.53	C-C4
E: *α*-Gal*p*A-(1→	5.07	76.53	C-C4
F: →6)-*α*-Glc*p*-(1→	4.90	78.74/65.58	B-C4/F-C6
G: →5)-*α*-Ara*f*-(1→	5.11	76.53	C-C4

## Data Availability

The original contributions presented in the study are included in the article/Supplementary Material, further inquiries can be directed to the corresponding author.

## Supplementary Materials

The following supporting information can be downloaded at: https://www.mdpi.com/article/10.3390/antiox15091056/s1, Figure S1: The elution profiles of polysaccharides fractions from SBG (A); Monosaccharide composition analysis (B). Figure S2 GC-MS total ion chromatogram of methylation analysis: NaBH_4_ (A); NaBD_4_ (B); Secondary mass spectrometry of glycosidic residues: T-linked-Ara*f* (C); T-linked-Gal*p*A (D); 1,5-linked-Ara*f* (E); 1,3-linked-Gal*p* (F); 1,3,5-linked-Ara*f* (G); 1,2,4-linked-Rha*p* (H); 1,4-linked-Gal*p* (I); 1,4-linked-Gal*p*A (J); 1,4-linked-Glc*p* (K); 1,2,5-linked-Ara*f* (L); 1,6-linked-Glc*p* (M); 1,3,4-linked-Gal*p*A (N); 1,2,3,5-linked-Ara*f* (O); 1,2,4-linked-Gal*p*A (P). Figure S3 Schematic representation of the experimental plan (A); Changes in body weight of ALI mice (B); Changes in lung index (C); The relative levels of protein (D), TNF-*α* (E), IL-6 (F), IL-1*β* (G), IL-4 (H), and IL-10 (I) in BALF; The relative levels of GSH (J), GSSG (K), GSH/GSSG (L), MDA (M), and SOD (N). Figure S4 Tax4Fun2-based functional prediction of differential genera. Figure S5 The relative abundance of *Lactobacillus johnsonii*.
