# Supplementary material for: Structural Characterization of Acidic Polysaccharides from Scutellaria baicalensis Georgi and Its Protection Against Acute Lung Injury
Source: Antioxidants (Basel). 2026 Aug 24;15(9):1056. doi: 10.3390/antiox15091056 (PMC13603109; doi:10.3390/antiox15091056)
Supplement: Supplementary file 1 [file antioxidants-15-01056-s001.zip › antioxidants-4470398-supplementary.pdf]

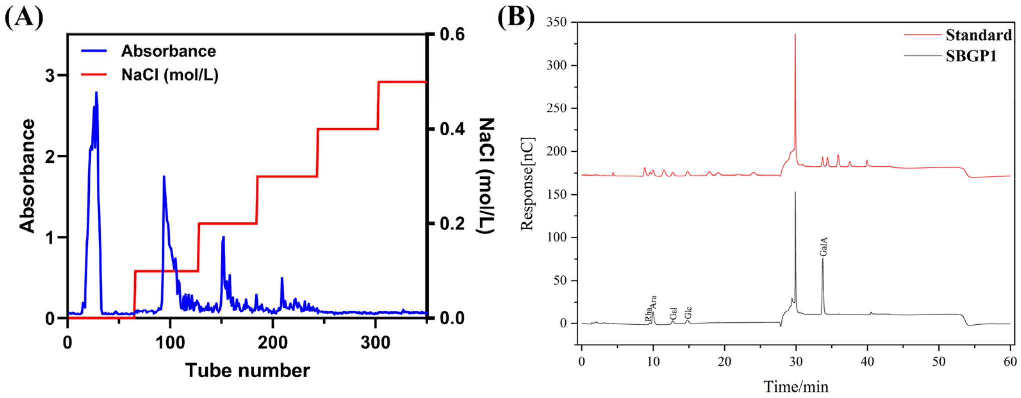

Figure S1 The elution profiles of polysaccharides fractions from SBG (A); Monosaccharide composition analysis (B).

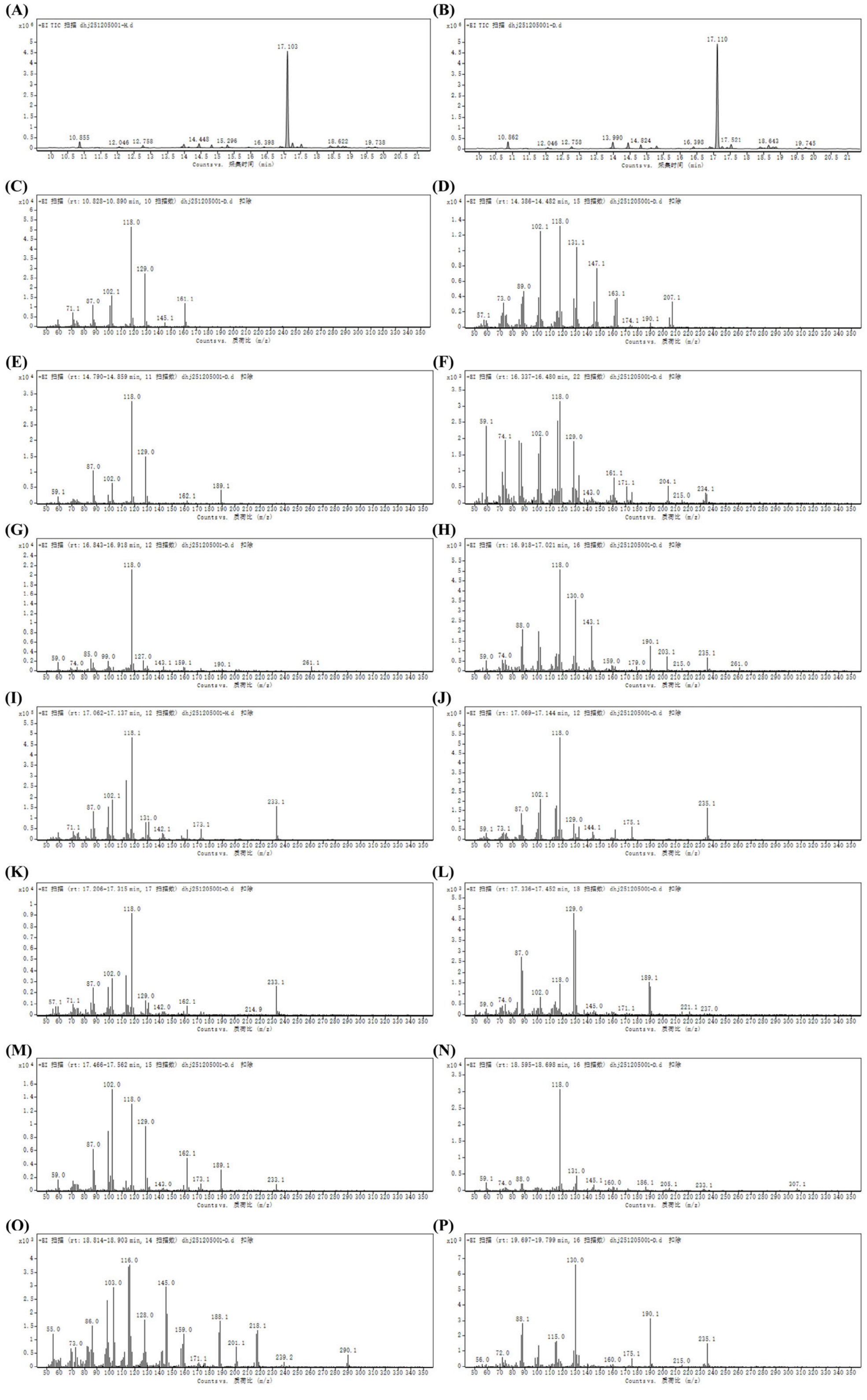

Figure S2 GC-MS total ion chromatogram of methylation analysis: $NaBH_4$ (A); $NaBD_4$ (B); Secondary mass spectrometry of glycosidic residues: T-linked-Ara*f* (C); T-linked-

Gal*p*A (D); 1,5-linked-Ara*f* (E); 1,3-linked-Gal*p* (F); 1,3,5-linked-Ara*f* (G); 1,2,4-linked-Rha*p* (H); 1,4-linked-Gal*p* (I); 1,4-linked-Gal*p*A (J); 1,4-linked-Glc*p* (K); 1,2,5-linked-Ara*f* (L); 1,6-linked-Glc*p* (M); 1,3,4-linked-Gal*p*A (N); 1,2,3,5-linked-Ara*f* (O); 1,2,4-linked-Gal*p*A (P).

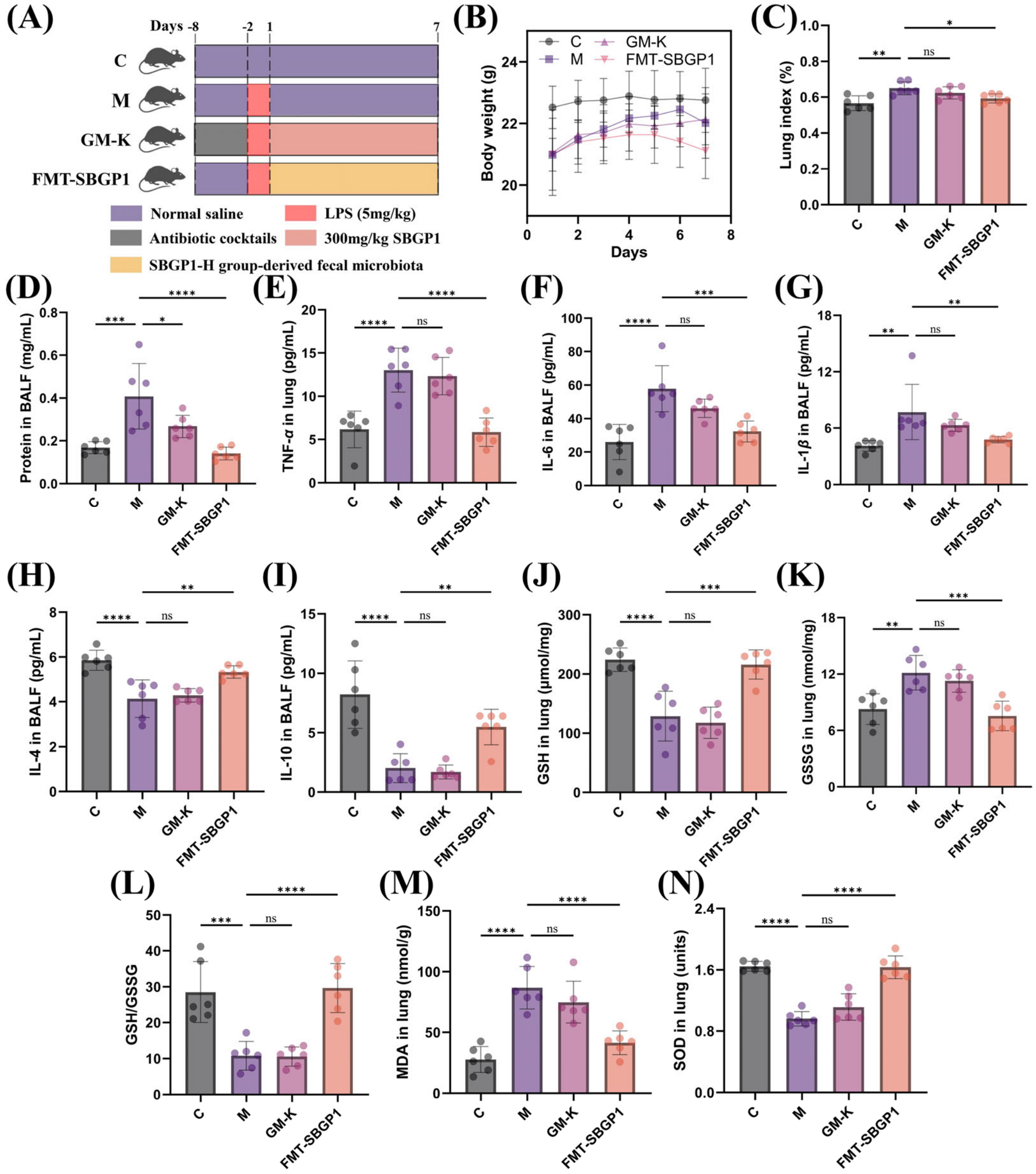

Figure S3 Schematic representation of the experimental plan (A); Changes in body weight of ALI mice (B); Changes in lung index (C); The relative levels of protein (D), TNF-α (E), IL-6 (F), IL-1β (G), IL-4 (H), and IL-10 (I) in BALF; The relative levels of GSH (J), GSSG (K), GSH/GSSG (L), MDA (M), and SOD (N). *$p < 0.05$, **$p < 0.01$, ***$p < 0.001$, and ****$p < 0.0001$.

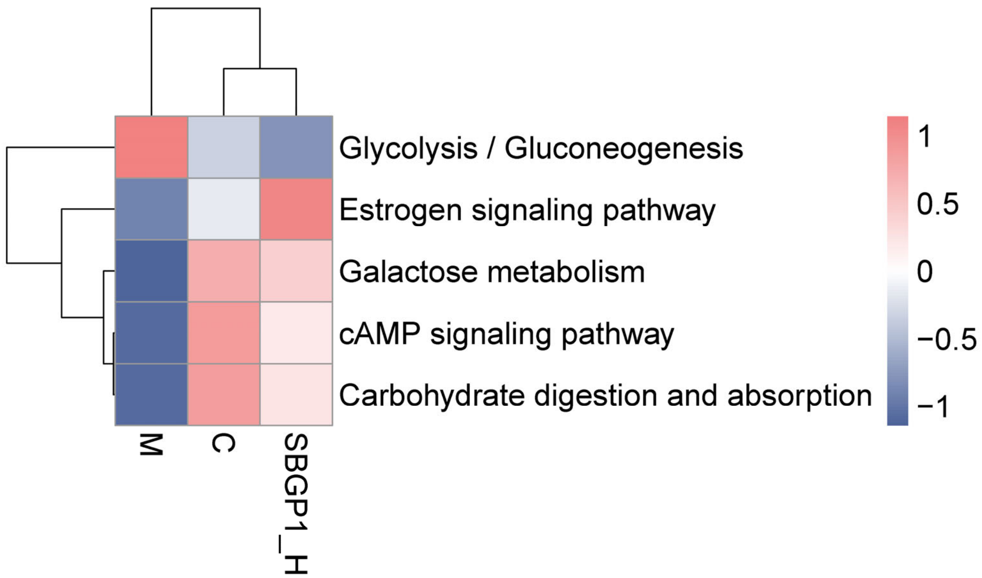

Figure S4 Tax4Fun2-based functional prediction of differential genera.

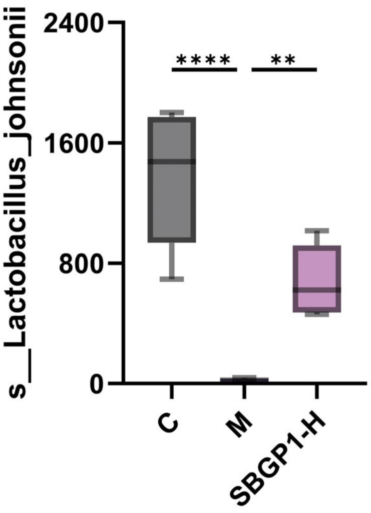

Figure S5 The relative abundance of *Lactobacillus johnsonii*. *$p < 0.05$, **$p < 0.01$, ***$p < 0.001$, and ****$p < 0.0001$.
